# Interface Contact Optimization via Phosphomolybdic Acid Enables 24.9% Efficiency in MoO_*X*_-Based Silicon Solar Cells

**DOI:** 10.1007/s40820-026-02250-4

**Published:** 2026-07-31

**Authors:** Shaopeng Chen, Qian Kang, Xiqi Yang, Hao Zhang, Jingjie Li, Wanyu Lu, Linfeng Yang, Tinghao Liu, Dayong Yuan, Zilong Zheng, Hui Yan, Yongzhe Zhang

**Affiliations:** 1 School of Information Science and Technology, Key Laboratory Optoelectronics Technology Ministry of Education, Beijing University of Technology, Beijing, 100124 People’s Republic of China; 2 School of Physics and Optoelectronic Engineering, Beijing University of Technology, Beijing, 100124 People’s Republic of China; 3 College of Materials Science and Engineering, Beijing University of Technology, Beijing, 100124 People’s Republic of China

**Keywords:** Interface engineering, Passivation, Silicon compound solar cells, MoO_*X*_, Hole transport layers

## Abstract

The development of cost-effective carrier-selective passivating contacts is critical for enhancing the commercial feasibility of silicon compound solar cells. Molybdenum oxide (MoO_*X*_) has garnered considerable interest as a promising hole transport layer (HTLs). A key advantage of MoO_*X*_ is high work function, in addition to the low-cost processability. However, in silicon photovoltaics, MoO_*X*_-based *p*-type contacts face fundamental limitations at hydrogenated amorphous silicon (i-a-Si:H)/MoO_*X*_ interface, where oxygen vacancy defects lower work function, as well as, weak van der Waals-dominated interactions impair charge carry transport. To address these challenges, we introduced an ultrathin phosphomolybdic acid (PMA) interlayer at the i-a-Si:H/MoO_*X*_ interface. PMA passivated oxygen vacancy defects, resulting in a notable improvement in open-circuit voltage from 713 to 730 mV, and 0.11 eV work function elevation via dipole formation; meanwhile, PMA strengthened the interfacial bonding energy, reducing saturation current density and contact resistance by 63% and 24%, respectively, contributing to a fill factor enhancement from 83.7% to 84.9%. In the end, we demonstrated a record efficiency of 24.9% for MoO_*X*_-based silicon solar cells, which provides valuable insights for developing high-performance MoO_*X*_ HTL devices for dopant-free *p*-type contact technologies.

**Supplementary Information:**

The online version contains supplementary material available at 10.1007/s40820-026-02250-4.

## Highlights

- We present a novel phosphomolybdic acid (PMA)/MoO_*X*_ that overcomes the intrinsic passivation and transport limitations of transition metal oxides in silicon photovoltaics.
- PMA acts as a dual-functional modifier, creating a strong interface dipole (6.34 D) and reducing the tunneling barrier, which lowers the contact resistance by 24%.
- The optimized dopant-free contact achieves a record-breaking efficiency of 24.9%, high-performance silicon solar cells.

**Supplementary Information:**

The online version contains supplementary material available at 10.1007/s40820-026-02250-4.

## Graphical abstract

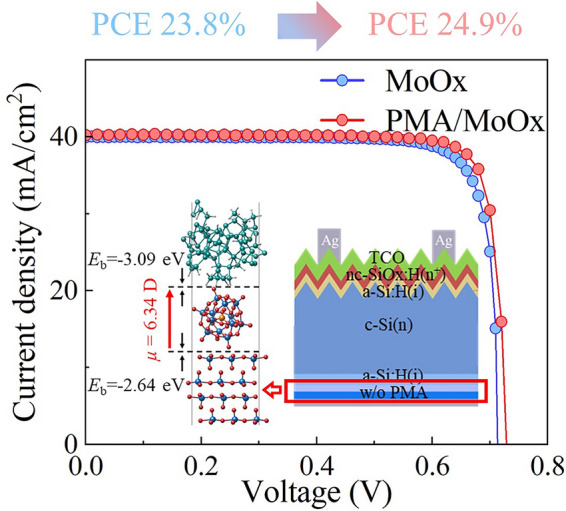

## Introduction

Crystalline silicon (c-Si) photovoltaics dominate over 95% of the solar market [1, 2], driving persistent research efforts to improve efficiency while reducing manufacturing costs [3, 4]. Achieving high efficiency requires superior surface passivation and carrier-selective layers. Conventional technologies, for instance, tunnel oxide passivating contacts (TOPCon) and silicon heterojunctions (SHJ) [5], employ *n*-/*p*-doped amorphous (a-Si), microcrystalline (μc-Si), or polycrystalline (poly-Si) silicon layers as electron/hole transport layers (ETLs/HTLs). However, these approaches require plasma-enhanced chemical vapor deposition (PECVD) equipment [6] and hazardous PH_3_/B_2_H_6_ doping gases [7, 8], and such doped layers introduce serious parasitic absorption and Auger recombination, ultimately limiting device performance [9].

Transition metal oxides have emerged as promising alternatives to address these challenges. For electron-selective contacts, materials such as magnesium oxide (MgO) [10], zinc oxide (ZnO) [11], and tantalum oxynitride (TaO_*X*_N_y_) [12] have been extensively explored. Their effectiveness relies on a low work function or a strong downward band bending at the c-Si interface, enabling efficient electron transport while blocking holes. Similarly, for hole-selective contacts, high-work function materials such as molybdenum oxide (MoO_*X*_) [13], tungsten oxide (WO_*X*_), vanadium oxide (V_2_O_*X*_), and nickel oxide (NiO_*X*_) [14] have been demonstrated to induce an inversion layer in crystalline silicon (c-Si), thereby facilitating efficient hole collection. These advancements further underscore the potential of dopant‑free passivating contact (DFPC) technology. DFPC refers to a contact scheme that achieves simultaneous surface passivation and carrier selectivity without extrinsic doping, and it can overcome the limitations of conventional doping processes.

Among them, MoO_*X*_ stands out as a leading hole transport layer (HTL), considering high work function (*W*_F_ > 5.5 eV), wide bandgap (*E*_g_ > 3.6 eV), and outstanding optical transparency (T > 90% across visible spectrum), as well as, compatibility with both solution-processing and thermal evaporation deposition techniques [15–17]. These advantages enable MoO_*X*_ suitable not only for hole-selective contacts in c-Si solar cells, but also for broader applications across various optoelectronic devices, for instance, organic photovoltaics (OPV) as an anode buffer layer, organic light-emitting diodes (OLEDs) and quantum dot LEDs (QLED) as a hole-injection layer [18–21].

A key distinction between MoO_*X*_-based and conventional *p*-doping-HTL SHJ (and TOPCon) lies in their hole-selectivity mechanism. SHJ (and TOPCon) relies on the drift transport of holes driven by the built-in electric field of *p*–*n* junction. In contrast, MoO_*X*_ contacts employ high work function engineering to achieve selective hole collection predominantly by diffusion transport. The work function difference between MoO_*X*_ and and *n*-type c-Si (*W*_F_ < 4.3 eV) induces strong band bending on the *n*-Si surface, creating a pronounced inversion layer that acts as a potential barrier to repel electrons while simultaneously accumulating holes at the interface potential well [22, 23]. Then, these accumulated holes transport to MoO_*X*_, through band-to-band or defect-assisted tunneling mechanisms, thereby achieving high-selective hole transport without conventional *p*-doping processes [15].

Despite these advantages, MoO_*X*_-based contacts in c-Si solar cells face fundamental challenges at the intrinsic hydrogenated a-Si (i-a-Si:H)/MoO_*X*_ interface [24]. Surface oxygen vacancy (V_O_) defects significantly reduce the work function of MoO_*X*_, while weak van der Waals-dominated interactions impair charge transport capability [25]. These limitations were reflected in the low open-circuit voltage (*V*_OC_ = 697 ~ 727 mV) of reported MoO_*X*_-based c-Si solar cells, which underperform compared to 750 mV of SHJ [26, 27].

In order to address these issues, we introduced phosphomolybdic acid (PMA, H_3_PMo_12_O_40_) at i-a-Si:H/MoO_*X*_ interface [28]. PMA is a metal–oxygen cluster compound, and previously used as an electrode buffer layer in organic electronics [29, 30], considering its soluble nature, and structural compatibility with MoO_*X*_. More importantly, PMA contains MoO_*X*_-like units and exhibits high work function, showing the potential to improve both energy level alignment and chemical bonding at i-a-Si:H/MoO_*X*_ interface. Thus, PMA can act as an interfacial bridge that enhances electronic and chemical properties of MoO_*X*_-based contact.

## Experiments and Methods

### Device Preparation

The composite crystalline silicon heterojunction solar cells with a rear-junction configuration were fabricated on *n*-type silicon substrates with a resistivity corresponding to a diameter range of 1.2–1.5 Ω cm and a thickness of 130 μm. The silicon wafers were cleaned and texturized via a wet chemical method. The brief process is as follows: First, the silicon wafer is immersed in a hot alkaline solution (a mixture of NaOH and isopropanol IPA) to form randomly distributed pyramidal structures with dimensions of approximately 1–3 μm. Subsequently, the wafer is cleaned sequentially using HCl solution and HF solution to remove metallic contaminants and the surface oxide layer, respectively, resulting in a clean textured silicon surface. Finally, the backside of the wafer is polished using a polishing slurry, yielding a silicon wafer with a textured front surface and a polished rear surface. For the front electron-selective contact stack, an 8-nm-thick i-a-Si:H layer was prepared by radio frequency plasma-enhanced chemical vapor deposition, followed by the deposition of a 15-nm-thick n-type nanocrystalline silicon oxide (*n*-nc-SiOx:H) layer using a high frequency plasma-enhanced chemical vapor deposition (PECVD) system. A transparent conductive oxide (TCO) layer, composed of traditional 10 wt% tin oxide-doped indium oxide (SnO_2_-doped In_2_O_3_), was deposited by magnetron sputtering. After printing the silver grid electrodes, annealing was performed at 190 °C for 30 min.

For PMA/MoO_*X*_ HTL (rear hole-selective contact), PMA was spin-coated using a spin coater (1500 rpm for 30 s), and then a 10-nm-thick MoO_*X*_ layer and a 200-nm-thick Ag layer were deposited by thermal evaporation, with rates of 0.1 and 1.5 Å s^−1^, respectively. The deposition pressure for both MoO_3_ powder and Ag particle sources was lower than 5 × 10^–4^ Pa.

### Characterization

A UV–visible spectrophotometer was used to measure the reflectance and absorbance of the films in the range of 300–1500 nm, and the optical band gap was determined using the Tauc equation. The elemental composition and chemical state of the films were characterized by X-ray photoelectron spectroscopy (XPS). This analysis utilized a monochromatic Al Kα X-ray source with a photon energy of 1486.7 eV, implemented on a Thermo Scientific ESCALab 250Xi measurement system. All acquired XPS spectra were processed and deconvoluted using Avantage software, employing a mixed Lorentz-Gaussian (GL) line shape for fitting. Additionally, all spectra were calibrated using the adventitious carbon C 1*s* peak at 284.8 eV as a reference. To determine the work function of the films, ultraviolet photoelectron spectroscopy (UPS) measurements were performed on the same ESCA Lab 250Xi system. The UPS spectra were excited using a He I ultraviolet resonance light source with a photon energy of 21.22 eV. The measurements were conducted with an energy resolution of < 120 meV and a step size of 0.02 eV over a scanning range of 0–20 eV. A negative sample bias of − 5 V was applied during the measurement to accurately collect the secondary electron cutoff signals. The film conductivity was measured by the two-probe method using a Keithley 2450 digital source meter and computationally fitted. The test samples were composed of Ag/*p*-Si/MoO_*X*_/Ag and Ag/*p*-Si/PMA/MoO_*X*_/Ag structures. A 200-nm layer of Ag was initially deposited onto a *p*-Si wafer. Subsequently, a mask with circular holes of different diameters was applied to define the area within which either MoOx was deposited. This was followed by another 200 nm of Ag through therma evaporation deposition. The contact resistivity (*ρ*_c_) was measured using the expanded Cox and Strack method (ECSM). More details on the calculation methods are provided in Supporting Information.

The *J-V* characteristics were measured under standard one-sun conditions (100 mW cm^−2^, AM 1.5G spectrum, 25 °C), with the light intensity calibrated using a certified reference cell from Fraunhofer Cal Lab. The external quantum efficiency (EQE) was obtained using a QE-R (Enlitech) measurement system. To evaluate the passivation quality, the effective minority carrier lifetime (*τ*_eff_) and the saturation current density (*J*_0_) of symmetrical test structures were measured using a Sinton WCT-120MX lifetime tester via the quasi-steady-state photoconductance (QSSPC) method. Physically, *τ*_eff_ is defined as the ratio of the average excess minority carrier density (Δ*n*_av_) to the total recombination rate (*R*), expressed as *τ*_eff_ = Δ*n*_av_/*R*. The symmetrical samples prepared for these measurements consisted of MoO_*X*_/i-a-Si:H/c-Si/i-a-Si:H/MoO_*X*_ and MoO_*X*_/PMA/i-a-Si:H/c-Si/i-a-Si:H/PMA/MoO_*X*_ stacks. The pseudo-illumination *J-V* curves were extracted via Suns-V_OC_ tests, specifically using the Suns-V_OC_ module of the Sinton WCT-120 instrument. The voltage changes of the devices were collected by reducing the light intensity of the flash lamp, which were then converted into Suns-V_OC_ curves. Capacitance–voltage (C–V) measurements were performed on complete device structures to investigate the depletion region characteristics and extract the built-in potential (*V*_bi_). The C–V sweeps were conducted using an electrochemical workstation for EIS measurements at an AC frequency of 10 kHz with an amplitude of 30 mV, while the DC bias was swept from − 0.05 to + 0.8 V. All measurements were performed at room temperature under dark conditions. The built-in potential was subsequently extracted from the *x*-intercept of the linear extrapolation of the Mott–Schottky plots (1/C^2^ versus voltage) in the reverse and low forward bias regions.

### Theoretical Simulations

First-principles calculations were performed based on density functional theory (DFT) as implemented in the Vienna Ab initio Simulation Package (VASP) [31, 32]. The electron-ion interactions were described using the projector augmented wave (PAW) method, while the exchange–correlation functional was treated with the standard Perdew–Burke–Ernzerhof (PBE) flavor of the generalized gradient approximation (GGA) [33, 34]. To account for long-range weak interactions within the systems, Grimme’s DFT-D3 scheme for dispersion corrections was applied in all calculations [35]. The kinetic energy cutoff is 500 eV for the expansion of the wavefunctions. All structural optimizations were conducted using the conjugate gradient (CG) algorithm until the residual forces on each unconstrained atom were less than 0.02 eV Å^−1^, with an electronic energy convergence threshold of 1 × 10^–5^ eV. For all slab models, the Brillouin zone was sampled using a Gamma-centered 3 × 3 × 1 Monkhorst–Pack k-point grid [36].

Energy band simulations were performed using the SCAPS-1D software package (version 3.3.07), a solar cell simulation program developed at the University of Gent, to conduct one-dimensional numerical analysis. The energy band alignment diagram under illumination was obtained by solving the Poisson equation and the continuity equations for electrons and holes. In the simulation, the effect of the PMA layer was treated as an equivalent modification of the interface boundary conditions. All material parameters were adopted from established literature and are summarized in Table S2. Simulations were performed at 300 K under AM 1.5G illumination to reflect the device operating conditions. By integrating the experimentally measured work function (5.11 eV, determined by UPS) with these semiconductor parameters, the simulation accurately captures the more pronounced upward band bending at the n-Si surface induced by the high-work function HTL and the corresponding enhancement in field-effect passivation.

Device simulations of c-Si solar cells incorporating MoO_*X*_ HTL were carried out using Silvaco TCAD software. The numerical modeling solved a set of fundamental semiconductor equations, including Poisson’s equation, carrier continuity equations, and drift–diffusion equations. All simulation parameters, provided in Tables S2 and S4, were taken from previously published studies and the Silvaco database [21, 27]. The refractive index and absorption coefficients for MoO_*X*_ were adopted from the literature [37]. The device simulations were performed at 300 K and incorporated multiple physical models, including Auger recombination, Shockley–Read–Hall (SRH) recombination, band-to-band (B2B) tunneling, direct tunneling, and thermal emission. Using the measured external quantum efficiency (EQE) data in conjunction with Quokka3’s T_ext_-Z function, we assisted the fitting process of the simulated solar cell parameters. All simulation parameters were taken from Ref. [38].

## Results and Discussion

### Device Performance and Optical Properties

To construct the PMA-incorporated device, we started from a conventional SHJ half-cell with structure of Ag/TCO/*n*-nc-SiO_*X*_:H/i-a-Si:H/c-Si/i-a-Si:H. Following hydrofluoric acid (HF) cleaning, ultrathin phosphomolybdic acid (PMA) layer was deposited by spin-coating onto i-a-Si:H surface. Subsequently, MoO_*X*_/Ag contact was formed by thermal evaporation (Fig. S1).

To determine the optimal PMA concentration, devices were prepared using PMA methanol solutions with concentrations ranging from 0.5 to 10 mg mL^−1^. Contact angle measurements confirmed the excellent wettability of the PMA solution on the i-a-Si:H surface (Fig. S2). The current density–voltage (*J*–*V*) characteristics revealed that the power conversion efficiency (*PCE*) initially increased and then decreased with increasing PMA concentration; A concentration of 2 mg mL^−1^ was identified as optimal, yielding the highest *PCE*, short-circuit current density (*J*_SC_), and fill factor (*FF*) (Fig. S3 and Table S1).

**Fig. 1:**
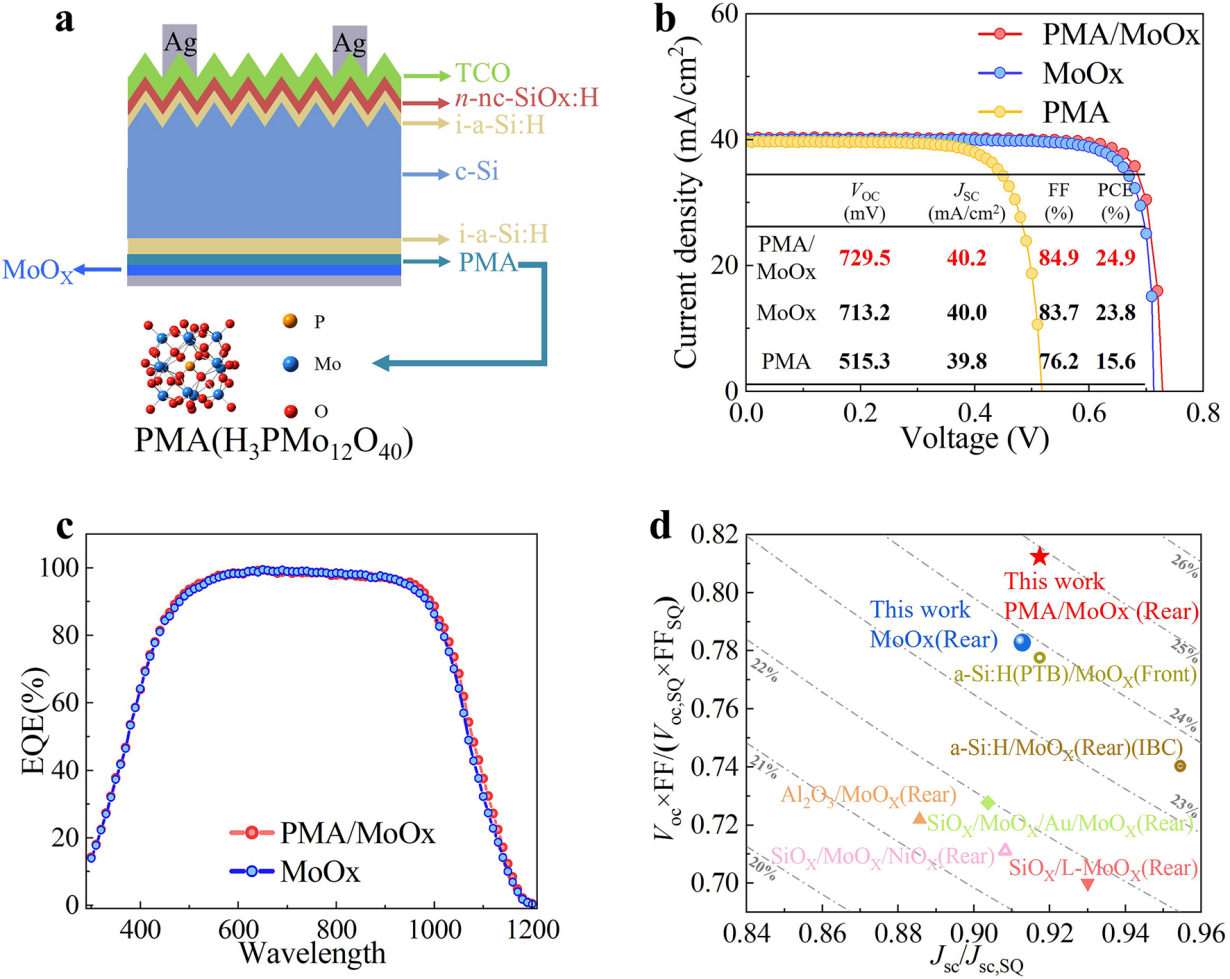
Device architecture and photovoltaic performance of c‑Si solar cells with MoO_X_, PMA/MoO_X_, and PMA HTLs.** a** Device structures of Ag/TCO/*n*-nc-SiO_X_:H/i-a-Si:H/c-Si/i-a-Si:H/HTL/Ag. The HTL is comprised of either MoO_X_, PMA and PMA/MoO_X_. The inset shows the molecular structure of PMA. **b** Experimental J‑V curves of control devices with MoO_X_, devices with PMA, and champion device with PMA/MoO_X_. **c** EQE spectra for devices with MoO_X_ and PMA/MoO_X_ HTLs. **d** Comparison of the electrical performance (*V*_OC_ × FF, normalized to the Shockley-Queisser limit, SQ) against the optical performance (*J*_SC_, normalized to SQ) for this work and previously reported Si composite heterojunction solar cells. The champion device in this work (red star) achieves a superior electrical performance value of 0.81 [21, 39–43]

Figure 1a illustrated the device structure, comparing the MoO_*X*_ HTL with the novel PMA/MoO_*X*_. The corresponding *J*–*V* curves are presented in Fig. 1b. The control device with MoO_*X*_ or PMA achieved PCEs of 23.8% and 15.6%. In contrast, the device using PMA/MoO_*X*_ exhibited a significant enhancement, with *V*_OC_ increasing from 713 to 730 mV and *FF* from 83.7% to 84.9%, culminating in a champion *PCE* of 24.9%. It is worth noting that MoO_*X*_ plays a dominant role in passivation compared to the device with only PMA.

The optical transmission properties of PMA/MoO_*X*_ and MoO_*X*_ films were characterized (Fig. S5). Both films exhibited high transmittance (over 95%) across the visible light range (400–800 nm). The optical bandgaps (*E*_g_) of the films, determined from Tauc plots [44], were found to be 3.63 eV for PMA/MoO_*X*_ and 3.60 eV for MoO_*X*_, respectively. Derived from Tauc equation, the *E*_g_ were determined to be of and films are 3.63 eV for PMA/MoO_*X*_ and 3.60 eV for MoO_*X*_, see the inset of Fig. S5, which is consistent with reported values for MoO_*X*_ and indicative of the wide bandgap nature of both materials [45]. The minimal difference in E_g_ confirms that incorporating the PMA interlayer does not markedly alter the optical band structure of HTL.

This conclusion is further corroborated by the highly similar external quantum efficiency (EQE) spectra (Fig. 1c) and the consistent *J*_SC_ values (Fig. 1b) observed for devices with and without PMA. The optimized device shows a marginally higher integrated *J*_SC_ (Fig. S4). The slightly higher *J*_SC_ in the PMA-modified device indicates that the PMA interlayer introduces negligible optical losses. Furthermore, it is worth noting that the direct contact between the MoO_*X*_ layer and the Ag rear electrode in our planar device architecture could potentially induce the excitation of surface plasmon polaritons (SPPs) at the metal–dielectric interface. As discussed in recent studies [46–49], these SPP modes can influence the optical field distribution, potentially contributing to near-field absorption or parasitic losses in the long-wavelength region. However, given that the *J*_SC_ showed only a marginal increase, the primary driver for maintaining such a high current is attributed to the improved electrical collection efficiency. The significant enhancements in *V*_OC_ and *FF* (resulting from the PMA-induced chemical and field-effect passivation) imply a substantial reduction in interfacial recombination velocity. This suppressed recombination ensures that photogenerated carriers, particularly those generated near the rear surface, are collected more efficiently rather than recombining, thereby maintaining a high *J*_SC_ despite the unoptimized rear reflector.

Finally, the champion PMA/MoO_*X*_ device was evaluated against reported Si composite heterojunction solar cells using the normalized performance *V*_OC_ × FF/(*V*_OC,SQ_ × FF_SQ_) and *J*_SC_/*J*_SC,SQ_ (Fig. 1d). The device delivered a superior electrical performance *V*_OC_ × FF/(*V*_OC,SQ_ × FF_SQ_) with value of 0.81, ranking among the highest reported result for Si composite heterojunction solar cells [21, 39–41, 43, 50].

### Charge Transport and Interfacial Mechanism

**Fig. 2:**
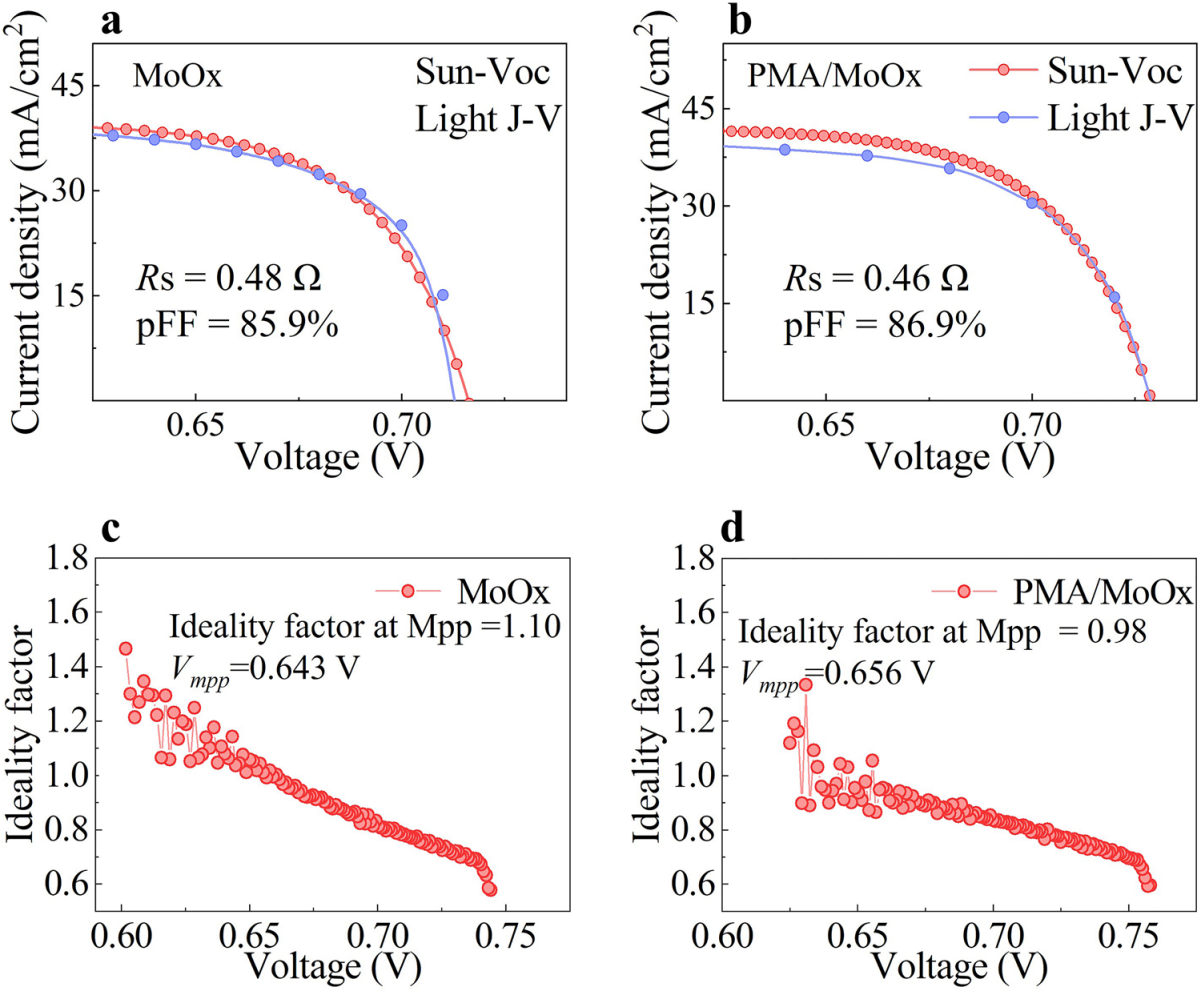
Electrical properties of Si heterojunction solar cells with PMA/MoO_X_ and MoO_X_ HTLs. Experimental light *J*–*V* curves and pseudo *J*–*V* from Suns-V_OC_ measurements for **a** MoO_X_ and **b** PMA/MoO_X_-based devices. Key electrical parameters are provided in the illustration. Voltage-dependent ideality factors (*n*) derived from the Suns-V_OC_ data for **c** MoO_X_ and **d** PMA/MoO_X_-based devices. The PMA/MoO_X_ device exhibits a consistently lower ideality factor across the measured voltage range, with a value of *n* = 0.98 at the maximum power point (*V*_mpp_), compared to *n* = 1.10 for MoO_X_ control, indicating significantly suppressed recombination losses and a more efficient carrier transport mechanism

To elucidate the origin of the improved *V*_OC_ and *FF*, we performed Suns-V_OC_ measurements. The difference between the light *J*–*V* curve and pseudo *J*–*V* curve (from Suns-V_OC_) yields the series resistance (*R*_*S*_), as shown in Fig. 2a, b. The device with the PMA interlayer showed a lower *R*_*S*_ (0.46 Ω cm^2^) than the control device (0.48 Ω cm^2^). More importantly, the pseudo fill factor (pFF), which reflects the intrinsic junction quality free from resistive losses [51], increased from 85.9% to 86.9% with PMA modification. AFM measurements (Fig. S6) show that the PMA/MoOX film exhibits a smoother surface compared with the MoO_*X*_-only film, favoring a more uniform contact and helping to suppress local shunting or high-field pathways.

This shows that the improvements in *V*_OC_ and *FF* stem not only from a reduction in *R*_S_, but also from effective suppression of interfacial non-radiative recombination. The light intensity-dependent *V*_OC_ (0.01–1 sun) of the devices is shown in Fig. S7. The excellent linearity observed for the PMA/MoO_*X*_ device indicates reduced leakage current and suppressed interfacial recombination under low-injection conditions.

The recombination pathways were further probed by analyzing the dependence of *V*_OC_ and light intensity and deriving the ideality factors (*n*) [52, 53] (Fig. 2c, d). The parameter n indicates whether carry transport is dominated by ideal diffusion (n ≈ 1) or by recombination (*n* ≈ 2) The PMA/MoO_*X*_-based device exhibit a more linear relationship between *V*_OC_ and illumination (Fig. S7), suggesting suppressed shunt leakage and better defect passivation under low injection [54]. Notably, the ideality factor at the voltage of the maximum power point (*V*_mpp_) was significantly reduced from 1.10 for the MoO_*X*_ device (*V*_mpp_ = 0.643 V) to 0.98 for the PMA/MoO_*X*_ device (*V*_mpp_ = 0.656 V). In high-efficiency silicon solar cells, an ideality factor dropping below unity indicates a transition in the dominant recombination mechanism [55]. Physically, as the PMA interlayer significantly suppresses the defect-mediated SRH recombination (*n* ≥ 1) at the interface, the intrinsic Auger recombination of the silicon bulk–which theoretically approaches an ideality factor of *n* = 2/3 at high injection levels–begins to manifest and pull the global ideality factor below 1 [55]. This reduction in the ideality factor firmly corroborates that PMA successfully minimizes interfacial recombination losses, allowing the device to operate closer to its fundamental efficiency limit.

**Fig. 3:**
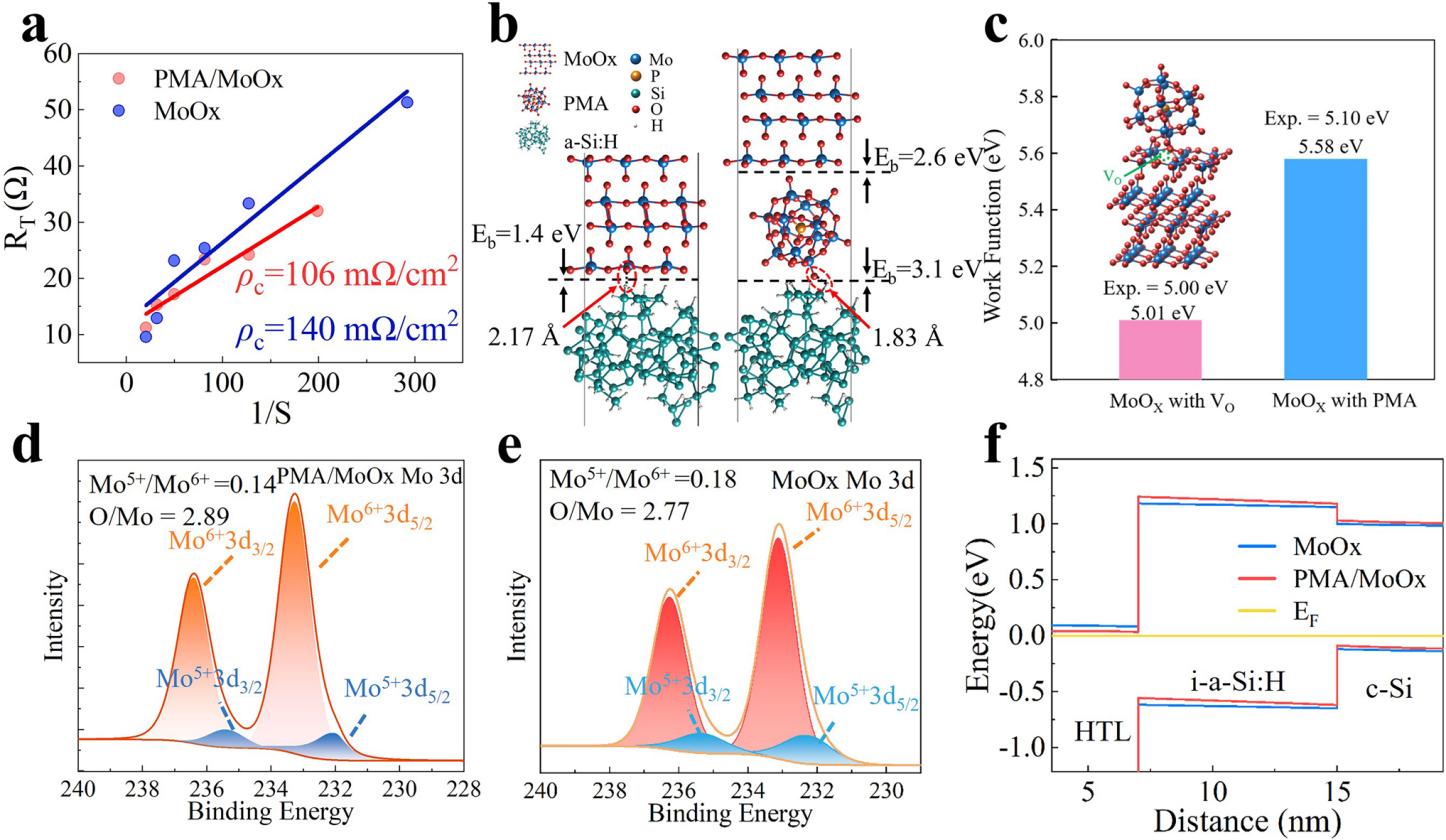
Interface analysis and mechanism of PMA/MoO_X_ HTL optimization. **a** Total resistance (*R*_*T*_) as a function of the inverse electrode area (1/S) for MoO_X_/i-a-Si:H/c-Si and MoO_X_/PMA/i-a-Si:H structures. **b** Equilibrium configurations of MoO_X_/i-a-Si:H and MoO_X_/PMA/i-a-Si:H interfaces obtained from first-principles calculations. **c** DFT-calculated *W*_F_ maps of MoO_X_ and PMA/MoO_X_ hole transport layer surfaces. **d, e** Mo 3*d* XPS spectra of the PMA/MoO_X_ and MoO_X_ thin film, showing a decrease in the Mo^5+^/Mo^6+^ ratio from 0.18 to 0.14 with PMA, confirming oxygen vacancy defect passivation. **f** Energy band diagrams simulated using SCAPS software, illustrating enhanced band bending with the PMA interlayer, which strengthens field-effect passivation and promotes hole transport

Cross-sectional TEM and STEM‑EDX mapping (Fig. S8) confirm that the PMA forms a continuous, ~ 1 nm‑thick interlayer on the i‑a‑Si:H surface. This molecular‑scale film ensures uniform coverage while being thin enough to allow efficient carrier tunneling. The contact resistance (*ρ*_c_) was quantified using the expanded Cox and Strack method (ECSM, schematic in Fig. S9) [56]. The application of the ECSM method is detailed in the Supporting Information, with the calculation fitting process as shown in Figs. S10 and S11. As shown in Fig. 3a, *ρ*_c_ was reduced by 24%, from 140 to 106 mΩ cm^2^ by incorporating the PMA interlayer, thus explaining the reduction in *R*_*S*_ and enhancement in *FF*. This shows that the improvements in *V*_OC_ and *FF* stem not only from a reduction in *Rs*, but also from effective suppression of interfacial non-radiative recombination. Consequently, the observed *FF* enhancement from 83.7 to 84.9% is attributed to the synergistic effect of reduced contact resistance and suppressed interfacial non-radiative recombination.

First-principles calculations were conducted to understand the atomistic mechanism behind the interface enhancement. The bonding energy and interfacial distance for MoO_*X*_/i-a-Si:H interface were calculated to be 1.4 eV and 2.5 Å, respectively, indicative of weak physical adsorption (Fig. 3b). The Keggin structure of PMA, with its protruding terminal oxygen atoms, facilitates better geometric matching and stronger chemical bonding (3.1 eV) with the i-a-Si:H surface. A large binding energy of 2.6 eV was also formed at MoO_*X*_/PMA interface, attributed to strong electrostatic and ionic interactions, creating a stable bridge for hole transport. As shown in Fig. 3c, the first-principles calculated work function maps reveal that, compared with MoO_*X*_, the PMA/MoO_*X*_ surface exhibits higher work function values, indicating that PMA effectively increases the work function of the hole transport layer. This stronger chemical bonding facilitates enhanced orbital overlap between the i-a-Si:H surface and the PMA molecule, creating a more electronically coupled interface. This improved coupling provides a more transparent pathway for hole transport by effectively lowering the tunneling barrier, thus significantly reducing the contact resistance (*ρ*_c_).

X-ray photoelectron spectroscopy (XPS) was employed to analyze the chemical states. The Mo 3*d* spectra of showed a decrease in the Mo^5+^/Mo^6+^ ratios from 0.18 for MoO_*X*_ to 0.14 for PMA/MoO_*X*_ [41], indicating that PMA passivates the oxygen vacancy (V_O_) defects on the MoO_*X*_ surface. Since V_O_ are associated with Mo^5+^ states, their reduction should increase the work function (*W*_F_). This was confirmed by ultraviolet photoelectron spectroscopy (UPS) measurements, showing *W*_F_ increased from 5.0 eV for MoO_*X*_ to 5.1 eV for PMA/MoO_*X*_ (Fig. S12).

To further uncover the microscopic origin of the electronic structure modification, we quantitatively extracted the macroscopic interface dipole moment (*μ*) as illustrated in Fig. S13. The pristine MoO_*X*_/i-a-Si:H interface exhibits a relatively small dipole moment of 1.63 D, whereas the introduction of the highly electronegative PMA interlayer triggers significant interfacial charge transfer (ICT), drastically amplifying the dipole moment to 6.34 D. This intense dipole layer (Δ*μ* = + 4.71 D) pointing toward the hole transport layer provides the direct physical origin for the work function modification. According to fundamental electrostatic principles, such a strong interfacial dipole inevitably induces a potential step (ΔΦ) across the interface, which macroscopically manifests as a shift in the local vacuum level and consequently an alteration of the *W*_F_.

The energy band alignments simulated using SCAPS software are depicted in Fig. 3f (the complete band diagram is shown in Fig. S14). The incorporation of PMA interlayer was found to significantly enhance the band bending at the c-Si/HTL interface. This stronger band bending, driven by the increased *W*_F_, enhances field-effect passivation by more effectively repelling electrons and facilitating hole collection, thereby contributing to the observed *V*_OC_ improvement [57].

### Passivation Quality and Junction Characteristics

**Fig. 4:**
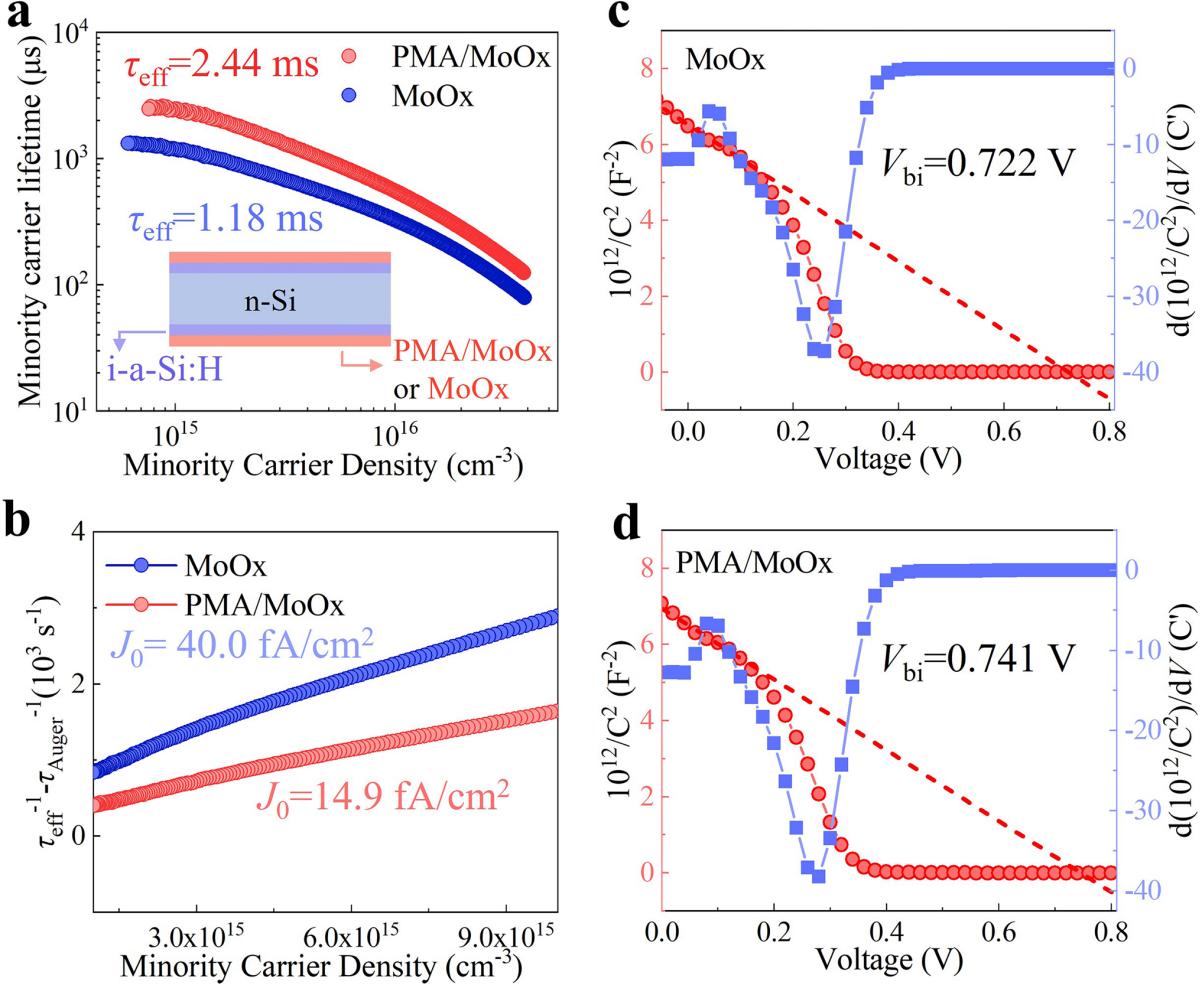
Quantification of passivation quality and junction characteristics. **a** Effective minority carrier lifetime (*τ*_eff_) as a function of injection level. **b** Reciprocal of the corrected effective lifetime (1/*τ*_eff_-1/*τ*_Auger_) [59] as a function of minority carrier concentration for extracting the saturation current density (*J*_0_). *τ*_Auger_ represents the carrier lifetime related to Auger recombination (see Supporting Information for the complete extraction expression). Mott-Schottky plots from C-V measurements for devices with **c** MoO_X_ and **d** PMA/MoO_X_ HTLs. The PMA/MoO_X_ device shows a higher built-in potential (*V*_bi_ = 0.741 V) than MoO_X_ device (*V*_bi_ = 0.722 V), confirming a stronger built-in electric field for improved charge extraction

The passivation was quantitatively assessed by measuring the effective minority carrier lifetime (*τ*_eff_), using quasi-steady-state photoconductance (QSSPC) method. The PMA/MoO_*X*_ sample exhibited a significantly higher *τ*_eff_ of 2.44 ms compared to 1.18 ms for the MoO_*X*_ sample, both measured at an excess carrier density of 1 × 10^15^ cm^−3^ (Fig. 4a). The saturation current density (*J*_0_) was extracted (Fig. 4b), revealing a substantial reduction from 40.0 fA cm^−2^ for the MoO_*X*_ contact to 14.9 fA cm^−2^ for the PMA/MoO_*X*_ contact. Furthermore, a comparison that includes pristine i-a-Si:H and PMA-only control samples (Fig. S15) shows that while the pristine i-a-Si:H layer yields the lowest *J*_0_, the PMA/MoO_*X*_ structure achieves the lowest *J*_0_ of 14.9 fA cm^−2^ among all evaluated transport layer configurations. This confirms the superior chemical passivation provided by the PMA interlayer within a functional contact stack that also enables efficient hole extraction. Capacitance–voltage (C–V) measurements were performed to analyze the junction properties [58] (Fig. 4c, d). The built-in potential (*V*_bi_) increased from 722 mV for the MoO_*X*_-based device to 741 mV for the PMA/MoO_*X*_-based device, consistent with the enhanced band bending observed in the simulations. The *V*_bi_ is determined by extrapolating the linear portion of the 1/C^2^-V plot to the voltage axis intercept. The stronger built-in electric field is crucial for efficient charge carrier separation and suppression of interface recombination.

### Quantifying Performance Enhancement via Simulation

To gain further insight into the influence of interfacial optimization on device performance, Quokka3 and Silvaco TCAD simulations were employed (Fig. S16). These two approaches provide complementary views of the interface improvement at different scales. Quokka3 evaluates the influence of interface-related recombination and contact parameters on the overall device output, whereas Silvaco TCAD describes the band structure and electrostatic changes associated with *W*_F_ modulation.

In the Quokka3 simulation, the experimentally extracted recombination *J*_0_ and *ρ*_c_ were introduced as input parameters (Fig. S16a, b). With the improved interface parameters, the simulated PCE increases from 23.9% to 24.7%. This result shows that the reduced interface recombination and improved contact transport are consistent with the enhanced device performance observed experimentally. Silvaco TCAD was then used to examine the effect of work function variation on the local energy band profile. Based on the UPS results, the increased work function leads to more favorable upward band bending at the c-Si surface (Fig. S16c, d), which is beneficial for carrier selectivity and interfacial carrier transport.

Overall, the two simulations provide complementary perspectives on the interface-related performance enhancement. Quokka3 connects the experimentally measured *J*_0_ and *ρ*_c_ values with the device-level output, while Silvaco TCAD illustrates how work function modulation affects the local electrostatic environment. These results suggest that the improved device performance is associated with reduced recombination/contact losses and favorable band structure modulation at the modified interface.

## Conclusion

In summary, we successfully developed a hole transport structure by a combination of phosphomolybdic acid (PMA) with the MoO_*X*_. The introduction of PMA interlayer between the hydrogenated amorphous silicon (i-a-Si:H) and MoO_*X*_ enabled a record power conversion efficiency of 24.9% for MoO_*X*_-based silicon compound solar cells, making a significant improvement from the control device efficiency of 23.6%. This enhancement was attributed to the notable increase in two key photovoltaic parameters, the open-circuit voltage (*V*_OC_) rose from 713 to 730 mV, and *FF* improved from 83.7 to 84.9%. The efficiency gains were driven by fundamental improvements in interfacial properties and electronic structure. PMA passivated oxygen vacancy (V_O_) defects on MoO_*X*_ surface, promoted favorable energy band bending via dipole formation, and elevated MoO_*X*_ work function by 0.11 eV. Furthermore, PMA strengthened the interfacial adhesion between i-a-Si:H and MoO_*X*_, leading to reduction in *J*_0_ from 40 to 15 fA cm^−2^ and interface contact resistance (*ρ*_c_) from 140 to 106 mΩ cm^2^. By addressing limitations of conventional MoO_*X*_-based contacts, including V_O_ defects and weak interfacial bonding, this interface engineering approach provided critical insights into the development of hole-selective contacts. The PMA/MoO_*X*_ composite structure provided a scalable [60] and cost-effective [24], manufacturing pathway for silicon photovoltaics, with promising potential for a broader application across various optoelectronic devices.

## Supplementary Information

Below is the link to the electronic supplementary material.

Supplementary file1 (DOCX 4.63 MB)

## References

[1] C. Ballif, F.-J. Haug, M. Boccard, P.J. Verlinden, G. Hahn, Status and perspectives of crystalline silicon photovoltaics in research and industry. Nat. Rev. Mater. **7**(8), 597–616 (2022). 10.1038/s41578-022-00423-2

[2] M.A. Green, The future of crystalline silicon solar cells. Prog. Photovolt. Res. Appl. **8**(1), 127–139 (2000). 10.1002/(SICI)1099-159X(200001/02)8:1<127::AID-PIP311>3.0.CO;2-D

[3] C. Battaglia, A. Cuevas, S. De Wolf, High-efficiency crystalline silicon solar cells: status and perspectives. Energy Environ. Sci. **9**(5), 1552–1576 (2016). 10.1039/c5ee03380b

[4] Z. Sun, X. Chen, Y. He, J. Li, J. Wang et al., Toward efficiency limits of crystalline silicon solar cells: recent progress in high-efficiency silicon heterojunction solar cells. Adv. Energy Mater. **12**(23), 2200015 (2022). 10.1002/aenm.202200015

[5] W. Li, Z. Xu, Y. Yan, J. Zhou, Q. Huang et al., Passivating contacts for crystalline silicon solar cells: an overview of the current advances and future perspectives. Adv. Energy Mater. **14**(18), 2304338 (2024). 10.1002/aenm.202304338

[6] N.L. Chang, G.K. Poduval, B. Sang, K. Khoo, M. Woodhouse et al., Techno-economic analysis of the use of atomic layer deposited transition metal oxides in silicon heterojunction solar cells. Prog. Photovolt. Res. Appl. **31**(4), 414–428 (2023). 10.1002/pip.3553

[7] N.S. Nath, I. Bhattacharya, A.G. Tuck, D.I. Schlipalius, P.R. Ebert, Mechanisms of phosphine toxicity. J. Toxicol. **2011**, 494168 (2011). 10.1155/2011/494168 21776261 10.1155/2011/494168 PMC3135219

[8] A.M. Monteiro, R.C. Flanagan, Process safety considerations for the use of 1 M borane tetrahydrofuran complex under general purpose plant conditions. Org. Process Res. Dev. **21**(2), 241–246 (2017). 10.1021/acs.oprd.6b00407

[9] X. Qu, Y. He, M. Qu, T. Ruan, F. Chu et al., Identification of embedded nanotwins at c-Si/a-Si: H interface limiting the performance of high-efficiency silicon heterojunction solar cells. Nat. Energy **6**(2), 194–202 (2021). 10.1038/s41560-020-00768-4

[10] H. Li, B. Gao, L. Yang, S. Wang, K. Gao et al., Magnesium oxide buffer layer for over 32% efficiency four-terminal perovskite/silicon tandem solar cells. Adv. Energy Mater. **15**(48), 2501762 (2025). 10.1002/aenm.202501762

[11] K. Gao, D. Xu, J. Wang, Q. Bi, Z. Wu et al., Efficient silicon solar cells with aluminum-doped zinc oxide-based passivating contact. Adv. Funct. Mater. **35**(4), 2415039 (2025). 10.1002/adfm.202415039

[12] W. Li, K. Gao, S. Wang, W. Shi, F. Cao et al., Sputtered tantalum oxynitride electron-selective contact for silicon solar cells. Appl. Phys. Lett. **126**(13), 133904 (2025). 10.1063/5.0269841

[13] K. Gao, Q. Bi, X. Wang, W. Liu, C. Xing et al., Progress and future prospects of wide-bandgap metal-compound-based passivating contacts for silicon solar cells. Adv. Mater. **34**(26), 2200344 (2022). 10.1002/adma.202200344 10.1002/adma.202200344 35524638

[14] Y. Wang, S.-T. Zhang, L. Li, X. Yang, L. Lu et al., Dopant-free passivating contacts for crystalline silicon solar cells: progress and prospects. EcoMat **5**(2), e12292 (2023). 10.1002/eom2.12292

[15] S. Cao, J. Li, J. Zhang, Y. Lin, L. Lu et al., Stable MoOX-based heterocontacts for p-type crystalline silicon solar cells achieving 20% efficiency. Adv. Funct. Mater. **30**(49), 2004367 (2020). 10.1002/adfm.202004367

[16] D. Scirè, P. Procel, A. Gulino, O. Isabella, M. Zeman et al., Sub-gap defect density characterization of molybdenum oxide: an annealing study for solar cell applications. Nano Res. **13**(12), 3416–3424 (2020). 10.1007/s12274-020-3029-9

[17] J. Meyer, S. Hamwi, M. Kröger, W. Kowalsky, T. Riedl et al., Transition metal oxides for organic electronics: energetics, device physics and applications. Adv. Mater. **24**(40), 5408–5427 (2012). 10.1002/adma.201201630 22945550 10.1002/adma.201201630

[18] H. Jinno, T. Yokota, M. Koizumi, W. Yukita, M. Saito et al., Self-powered ultraflexible photonic skin for continuous bio-signal detection via air-operation-stable polymer light-emitting diodes. Nat. Commun. **12**, 2234 (2021). 10.1038/s41467-021-22558-6 33854058 10.1038/s41467-021-22558-6 PMC8047008

[19] M. Riahi, K. Yoshida, I.D.W. Samuel, Improving the air stability of flexible top-emitting organic light-emitting diodes. npj Flex. Electron. **8**, 51 (2024). 10.1038/s41528-024-00338-8

[20] Y. Zhang, Y. Zhan, G. Yuan, X. Chen, X. Lu et al., Record high external quantum efficiency of 20% achieved in fully solution-processed quantum dot light-emitting diodes based on hole-conductive metal oxides. J. Colloid Interface Sci. **660**, 746–755 (2024). 10.1016/j.jcis.2024.01.099 38271810 10.1016/j.jcis.2024.01.099

[21] L. Cao, P. Procel, A. Alcañiz, J. Yan, F. Tichelaar et al., Achieving 23.83% conversion efficiency in silicon heterojunction solar cell with ultra-thin MoO_*x*_ hole collector layer *via* tailoring (i) a-Si: H/MoO_*x*_ interface. Prog. Photovoltaics **31**(12), 1245–1254 (2023). 10.1002/pip.3638

[22] T.G. Allen, J. Bullock, X. Yang, A. Javey, S. De Wolf, Passivating contacts for crystalline silicon solar cells. Nat. Energy **4**(11), 914–928 (2019). 10.1038/s41560-019-0463-6

[23] B.K. Mondal, S.K. Mostaque, M.A. Rashid, A. Kuddus, H. Shirai et al., Effect of CdS and In_3_Se_4_ BSF layers on the photovoltaic performance of PEDOT: PSS/n-Si solar cells: simulation based on experimental data. Superlattices Microstruct. **152**, 106853 (2021). 10.1016/j.spmi.2021.106853

[24] Q. Yao, S. Li, L. Dong, P. Gu, X. Liu et al., Achieving tunable work function in MoO thin films: a key to low-cost, high-performance organic electronics. Thin Solid Films **817**, 140659 (2025). 10.1016/j.tsf.2025.140659

[25] H. Cai, X. Yang, X. Xu, Q. Zeng, S. Zhou et al., Enhancing silicon compound heterojunction solar cells with vanadium-doped MoO_X_ as hole transport layers. Adv. Sci. **12**(28), 2505929 (2025). 10.1002/advs.202505929 10.1002/advs.202505929 PMC12302542 40344471

[26] Q. Gao, Z. Xu, Y. Yan, W. Li, Y. Song et al., Efficient hole transport layers for silicon heterojunction solar cells by surface plasmonic modification in MoOx/Au NPs/MoOx stacks. Mater. Today Energy **45**, 101681 (2024). 10.1016/j.mtener.2024.101681

[27] H. Lin, M. Yang, X. Ru, G. Wang, S. Yin et al., Silicon heterojunction solar cells with up to 26.81% efficiency achieved by electrically optimized nanocrystalline-silicon hole contact layers. Nat. Energy **8**(8), 789–799 (2023). 10.1038/s41560-023-01255-2

[28] J. Lv, Z. Li, R. Dong, Y. Xue, Y. Wang et al., Highly flame-retardant materials of different divalent metal ions alginate/silver phosphate: synthesis, characterizations, and synergistic phosphorus-polymetallic effects. Int. J. Biol. Macromol. **247**, 125834 (2023). 10.1016/j.ijbiomac.2023.125834 37453641 10.1016/j.ijbiomac.2023.125834

[29] L. Feng, Z. Li, Y. Liu, L. Hua, Z. Wei et al., Counterion engineering toward high-performance and pH-neutral polyoxometalates-based hole-transporting materials for efficient organic optoelectronic devices. ACS Nano **18**(4), 3276–3285 (2024). 10.1021/acsnano.3c09865 38252155 10.1021/acsnano.3c09865

[30] L.C. Palilis, M. Vasilopoulou, A.M. Douvas, D.G. Georgiadou, S. Kennou et al., Solution processable tungsten polyoxometalate as highly effective cathode interlayer for improved efficiency and stability polymer solar cells. Sol. Energy Mater. Sol. Cells **114**, 205–213 (2013). 10.1016/j.solmat.2013.02.034

[31] G. Kresse, J. Furthmüller, Efficiency of ab-initio total energy calculations for metals and semiconductors using a plane-wave basis set. Comput. Mater. Sci. **6**(1), 15–50 (1996). 10.1016/0927-0256(96)00008-0 10.1103/physrevb.54.11169 9984901

[32] J. Neugebauer, T. Hickel, Density functional theory in materials science. WIREs Comput. Mol. Sci. **3**(5), 438–448 (2013). 10.1002/wcms.1125 10.1002/wcms.1125 PMC3920634 24563665

[33] G. Lippert, J. Hutter, M. Parrinello, A hybrid Gaussian and plane wave density functional scheme. Mol. Phys. **92**(3), 477–487 (1997). 10.1080/00268979709482119

[34] J.P. Perdew, K. Burke, M. Ernzerhof, Generalized gradient approximation made simple. Phys. Rev. Lett. **77**(18), 3865–3868 (1996). 10.1103/physrevlett.77.3865 10062328 10.1103/PhysRevLett.77.3865

[35] S. Grimme, J. Antony, S. Ehrlich, H. Krieg, A consistent and accurate ab initio parametrization of density functional dispersion correction (DFT-D) for the 94 elements H-Pu. J. Chem. Phys. **132**(15), 154104 (2010). 10.1063/1.3382344 20423165 10.1063/1.3382344

[36] W. Setyawan, S. Curtarolo, High-throughput electronic band structure calculations: challenges and tools. Comput. Mater. Sci. **49**(2), 299–312 (2010). 10.1016/j.commatsci.2010.05.010

[37] A. Kumar, M. Dutta, S. Tomer, P. Rao, Vandana et al., Tuning of structural and optical properties of reactively sputtered MoOx films. J. Mater. Sci. Mater. Electron. **35**(8), 612 (2024). 10.1007/s10854-024-12324-x

[38] W. Lu, X. Yang, Q. Kang, J. Li, Z. Zhou et al., Patterning design for edge passivation in compound crystalline silicon solar cells. Adv. Funct. Mater. **36**(11), e14833 (2026). 10.1002/adfm.202514833

[39] J. Dréon, Q. Jeangros, J. Cattin, J. Haschke, L. Antognini et al., 23.5%-efficient silicon heterojunction silicon solar cell using molybdenum oxide as hole-selective contact. Nano Energy **70**, 104495 (2020). 10.1016/j.nanoen.2020.104495

[40] J. Li, Q. Kang, Y. Wang, Z. Zhou, Z. Sun et al., Low oxygen content MoO_x_ and SiO_x_ tunnel layer based heterocontacts for efficient and stable crystalline silicon solar cells approaching 22% efficiency. Adv. Funct. Mater. **34**(6), 2310619 (2024). 10.1002/adfm.202310619

[41] W. Lu, Q. Kang, J. Li, X. Xu, D. Yuan et al., MoOx/Au/MoOx-based composite heterocontacts for crystalline silicon solar cells achieving 22.0% efficiency. Small Struct. **6**(7), 2400559 (2025). 10.1002/sstr.202400559

[42] L. Li, G. Du, Y. Lin, X. Zhou, Z. Gu et al., NiOx/MoOx bilayer as an efficient hole-selective contact in crystalline silicon solar cells. Cell Rep. Phys. Sci. **2**(12), 100684 (2021). 10.1016/j.xcrp.2021.100684

[43] S. Chowdhury, M.Q. Khokhar, D.P. Pham, J. Yi, Al_2_O_3_/MoO_*x*_ hole-selective passivating contact for silicon heterojunction solar cell. ECS J. Solid State Sci. Technol. **11**(1), 015004 (2022). 10.1149/2162-8777/ac4d83

[44] J. Klein, L. Kampermann, B. Mockenhaupt, M. Behrens, J. Strunk et al., Limitations of the Tauc plot method. Adv. Funct. Mater. **33**(47), 2304523 (2023). 10.1002/adfm.202304523

[45] S. Erfanifam, S.M. Mohseni, L. Jamilpanah, M. Mohammadbeigi, P. Sangpour et al., Tunable bandgap and spin-orbit coupling by composition control of MoS_2_ and MoOx (x=2 and 3) thin film compounds. Mater. Des. **122**, 220–225 (2017). 10.1016/j.matdes.2017.02.085

[46] H. Nasser, M.Z. Borra, E.H. Çiftpınar, B. Eldeeb, R. Turan, Fourteen percent efficiency ultrathin silicon solar cells with improved infrared light management enabled by hole-selective transition metal oxide full-area rear passivating contacts. Prog. Photovolt. Res. Appl. **30**(8), 823–834 (2022). 10.1002/pip.3510

[47] H. Nasser, F. Es, M. Zolfaghari Borra, E. Semiz, G. Kökbudak et al., On the application of hole-selective MoOx as full-area rear contact for industrial scale p-type c-Si solar cells. Prog. Photovoltaics **29**(3), 281–293 (2021). 10.1002/pip.3363

[48] S. Zhong, J. Dreon, Q. Jeangros, E. Aydin, S. De Wolf et al., Mitigating plasmonic absorption losses at rear electrodes in high-efficiency silicon solar cells using dopant-free contact stacks. Adv. Funct. Mater. **30**(5), 1907840 (2020). 10.1002/adfm.201907840

[49] Z.C. Holman, M. Filipič, B. Lipovšek, S. De Wolf, F. Smole et al., Parasitic absorption in the rear reflector of a silicon solar cell: simulation and measurement of the sub-bandgap reflectance for common dielectric/metal reflectors. Sol. Energy Mater. Sol. Cells **120**, 426–430 (2014). 10.1016/j.solmat.2013.06.024

[50] Z. Liu, H. Lin, Z. Wang, L. Chen, T. Wu et al., Dual functional dopant-free contacts with titanium protecting layer: boosting stability while balancing electron transport and recombination losses. Adv. Sci. **9**(23), 2202240 (2022). 10.1002/advs.202202240 10.1002/advs.202202240 PMC9376810 35703126

[51] D. Kiermasch, L. Gil-Escrig, H.J. Bolink, K. Tvingstedt, Effects of masking on open-circuit voltage and fill factor in solar cells. Joule **3**(1), 16–26 (2019). 10.1016/j.joule.2018.10.016

[52] H. Lin, G. Wang, Q. Su, C. Han, C. Xue et al., Unveiling the mechanism of attaining high fill factor in silicon solar cells. Prog. Photovoltaics **32**(6), 359–371 (2024). 10.1002/pip.3775

[53] F. Khan, S.-H. Baek, J.H. Kim, Intensity dependency of photovoltaic cell parameters under high illumination conditions: an analysis. Appl. Energy **133**(C), 356–362 (2014). 10.1016/j.apenergy.2014.07.107

[54] R.H. Sardar, A. Bera, S. Chattopadhyay, S.I. Ali, S. Pramanik et al., The impact of series (R_s_) and shunt resistances (R_sh_) on solar cell parameters to enhance the photovoltaic performance of f-PSCs. Opt. Mater. **155**, 115818 (2024). 10.1016/j.optmat.2024.115818

[55] G. Wang, M. Yu, H. Wu, Y. Li, L. Xie et al., Silicon solar cells with hybrid back contacts. Nature **647**(8089), 369–374 (2025). 10.1038/s41586-025-09681-w 41224977 10.1038/s41586-025-09681-w

[56] L. Chen, H. Lin, Z. Liu, T. Wu, Y. Pang et al., Realization of a general method for extracting specific contact resistance of silicon-based dopant-free heterojunctions. Solar RRL **6**(2), 2100394 (2022). 10.1002/solr.202100394

[57] M. Vaqueiro-Contreras, C. Bartlam, R.S. Bonilla, V.P. Markevich, M.P. Halsall et al., Graphene oxide films for field effect surface passivation of silicon for solar cells. Sol. Energy Mater. Sol. Cells **187**, 189–193 (2018). 10.1016/j.solmat.2018.08.002

[58] J. Wu, Z. Ying, X. Li, M. Zhang, X. Guo et al., Surface sulfuration of atomic layer deposited SnOx for enhanced performance of n–i–P perovskite solar cells. Solar RRL **9**(7), 2400879 (2025). 10.1002/solr.202400879

[59] L. Li, G. Du, X. Zhou, Y. Lin, Y. Jiang et al., Interfacial engineering of Cu_2_O passivating contact for efficient crystalline silicon solar cells with an Al_2_O_3_ passivation layer. ACS Appl. Mater. Interfaces **13**(24), 28415–28423 (2021). 10.1021/acsami.1c08258 34120440 10.1021/acsami.1c08258

[60] R. Zhang, B. Ma, Y. Cui, C. Tan, B. Chen et al., Designing MoO_x_/Ag/MoO_x_ sandwich structured buffer layer for four-terminal CsPbI_3_/TOPCon tandem minimodules. Mater. Futur. **4**(4), 045103 (2025). 10.1088/2752-5724/ae0c76

