# Supplementary material for: Interface Contact Optimization via Phosphomolybdic Acid Enables 24.9% Efficiency in MoOX-Based Silicon Solar Cells
Source: Nanomicro Lett. 2026 Jul 31;19:16. doi: 10.1007/s40820-026-02250-4 (PMC13427687; doi:10.1007/s40820-026-02250-4)
Supplement: Supplementary file 1 — Supplementary file1 (DOCX 4.63 MB) [file 40820_2026_2250_MOESM1_ESM.docx]

Supporting Information for

**Interface Contact Optimization via Phosphomolybdic Acid Enables 24.9% Efficiency in MoO_X_-Based Silicon Solar Cells**

Shaopeng Chen^1^, Qian Kang^1,^*, Xiqi Yang^3^, Hao Zhang^3^, Jingjie Li^2^, Wanyu Lu^1^, Linfeng Yang^1^, Tinghao Liu^3^, Dayong Yuan^1^, Zilong Zheng^3,^*, Hui Yan^3^, and Yongzhe Zhang^1,^*

^1^ School of Information Science and Technology, Key Laboratory Optoelectronics Technology Ministry of Education, Beijing University of Technology, Beijing 100124, P. R. China

^2^ School of Physics and Optoelectronic Engineering, Beijing University of Technology, Beijing 100124, P. R. China

^3^ College of Materials Science and Engineering, Beijing University of Technology, Beijing 100124, P. R. China

*Corresponding authors. E-mail: [kangqian@bjut.edu.cn](mailto:kangqian@bjut.edu.cn) (Qian Kang); [zilong.zheng@bjut.edu.cn](mailto:zilong.zheng@bjut.edu.cn) (Zilong Zheng); [yzzhang@bjut.edu.cn](mailto:yzzhang@bjut.edu.cn) (Yongzhe Zhang)

**Supplementary Figures and Tables**


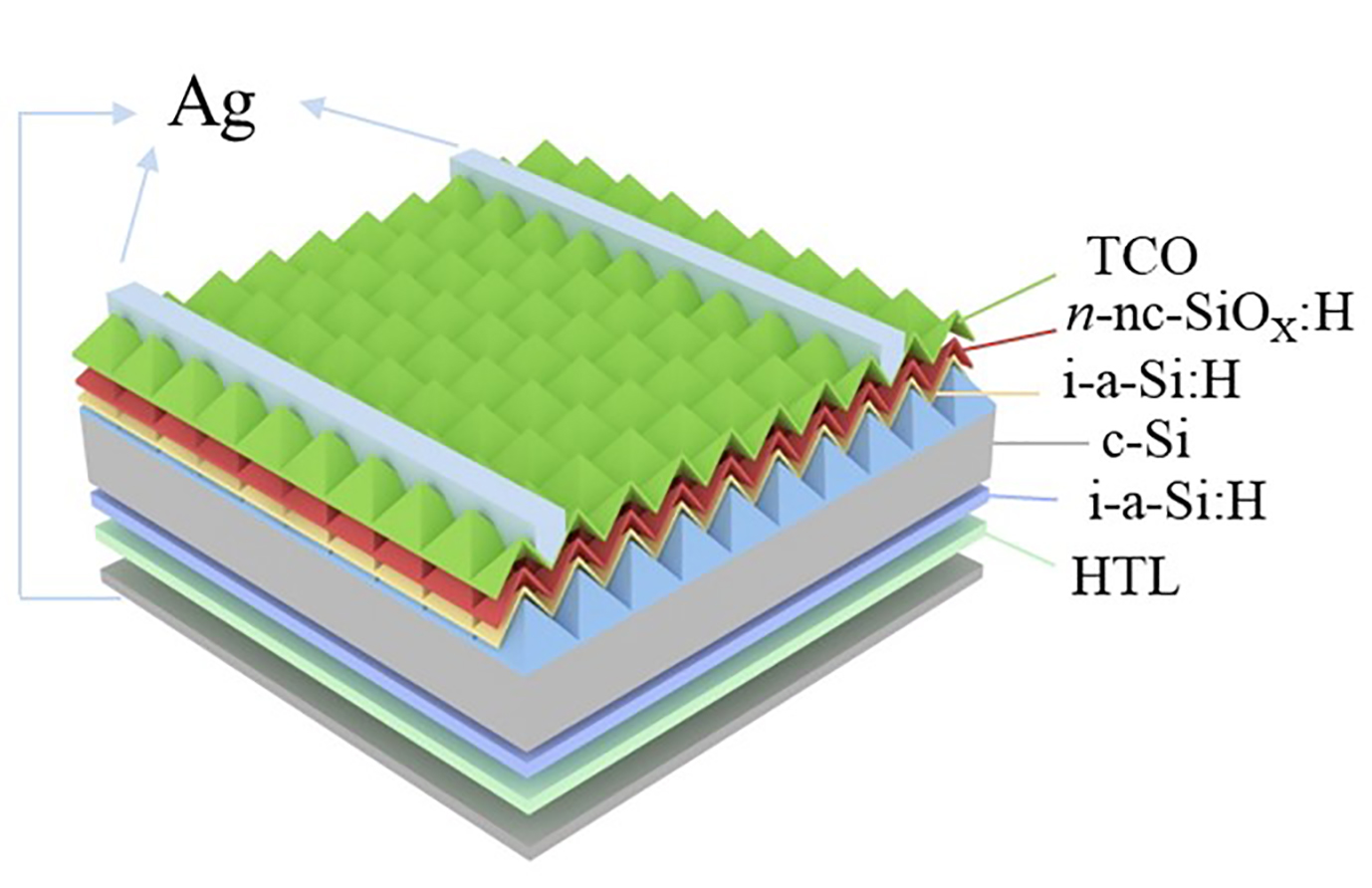


**Fig. S1** 3D structural schematic diagram of the Ag/TCO/*n*-a-Si:H/i-a-Si:H/n-Si/i-a-Si:H/HTL/Ag solar cell.


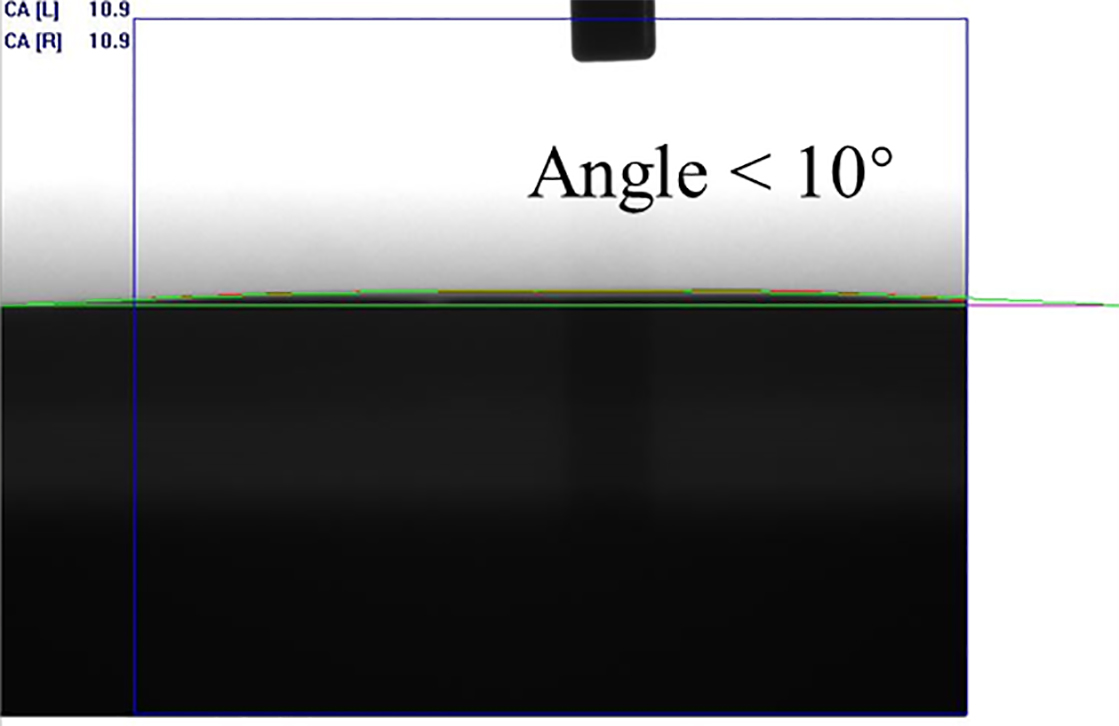


**Fig. S2** Contact angle (CA) images of PMA (ethanol) solution on i-a-Si:H.


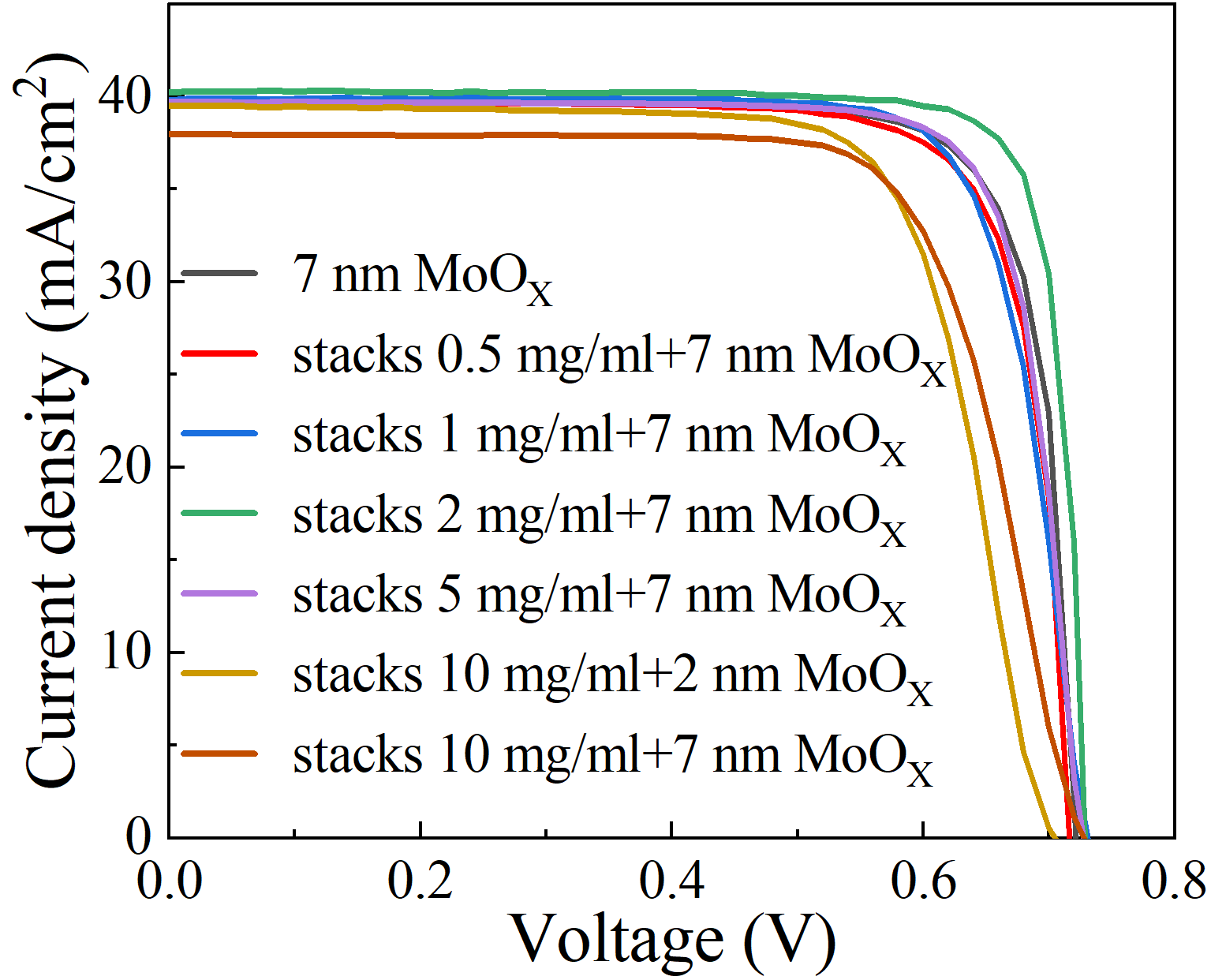


**Fig. S3** J-V curves of heterocontact solar cells with varied PMA concentrations (0.5-10 mg/ml) and MoO_X_ thickness (2 or 7 nm), under AM1.5G illumination.


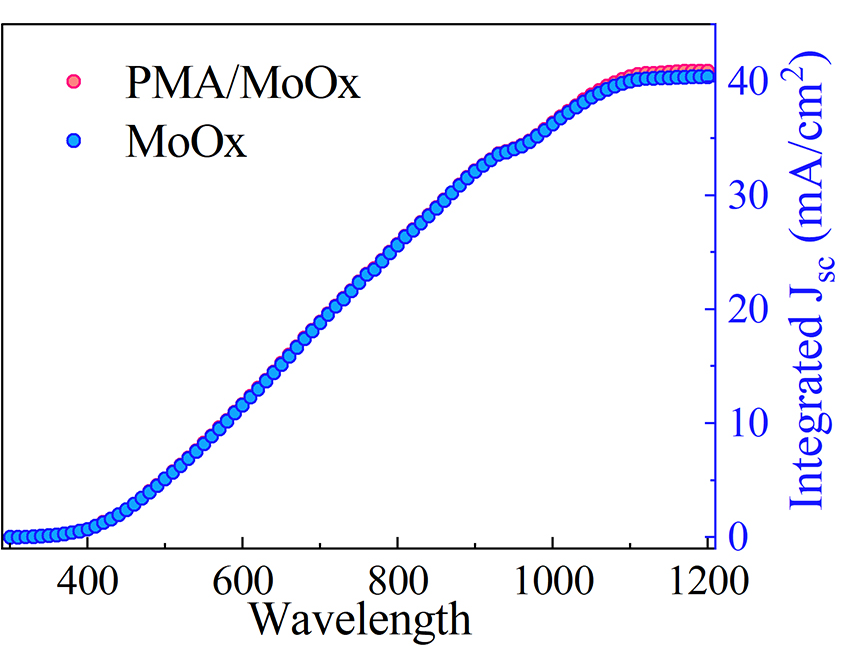
­

**Fig. S4** Integrated J_SC_ values of solar cells treated with MoO_X_ and PMA/MoO_X_ as the HTL. The higher integrated current of the PMA/MoO_X_ device aligns with its enhanced photo-response over 400-800 nm.


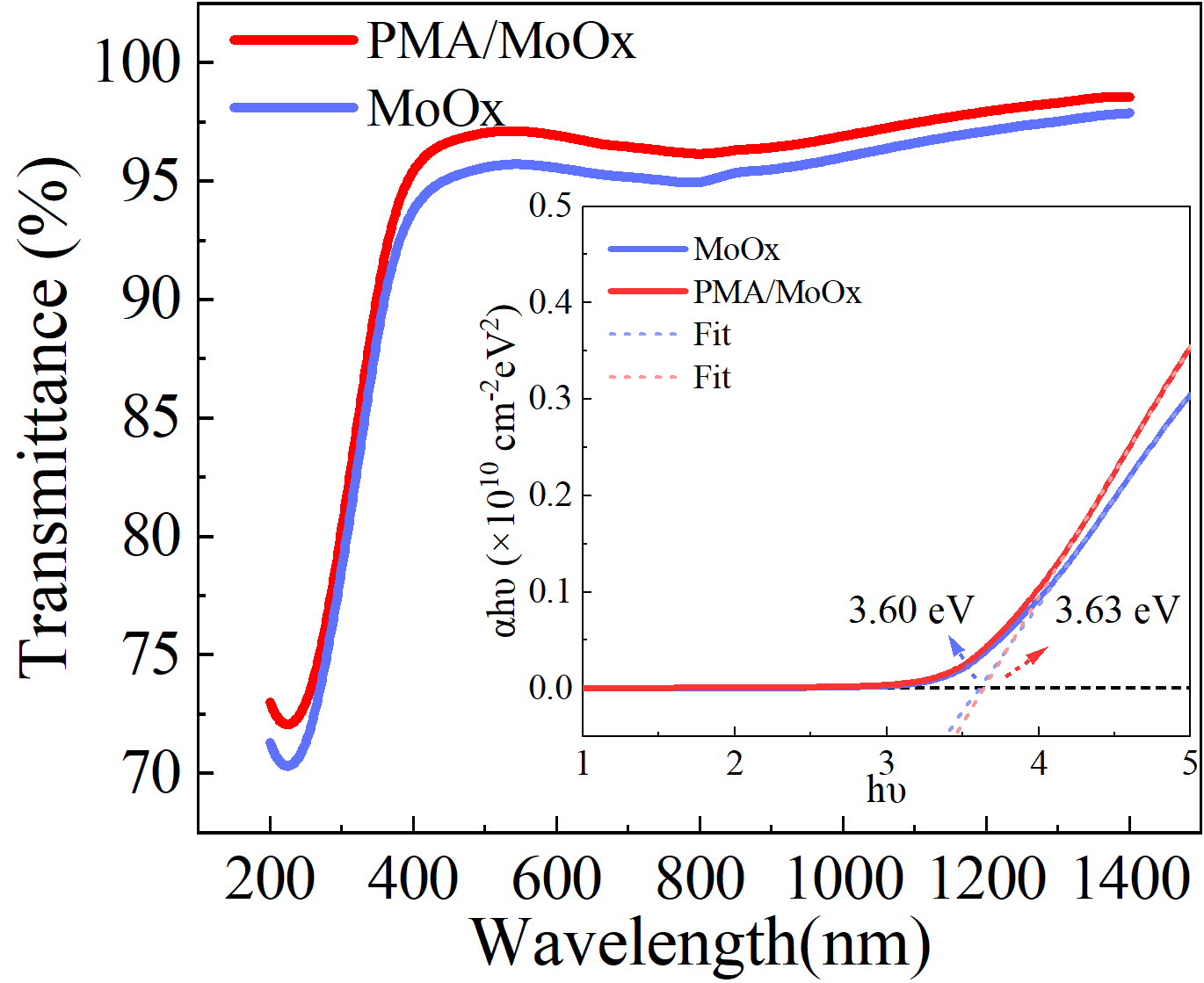


**Fig. S5** UV-vis absorption spectra and transmittance of PMA/MoO_X_ and MoO_X_ films. The inset showed the variation of (𝛼 h𝜐)^2^ with the photon energy h𝜐.


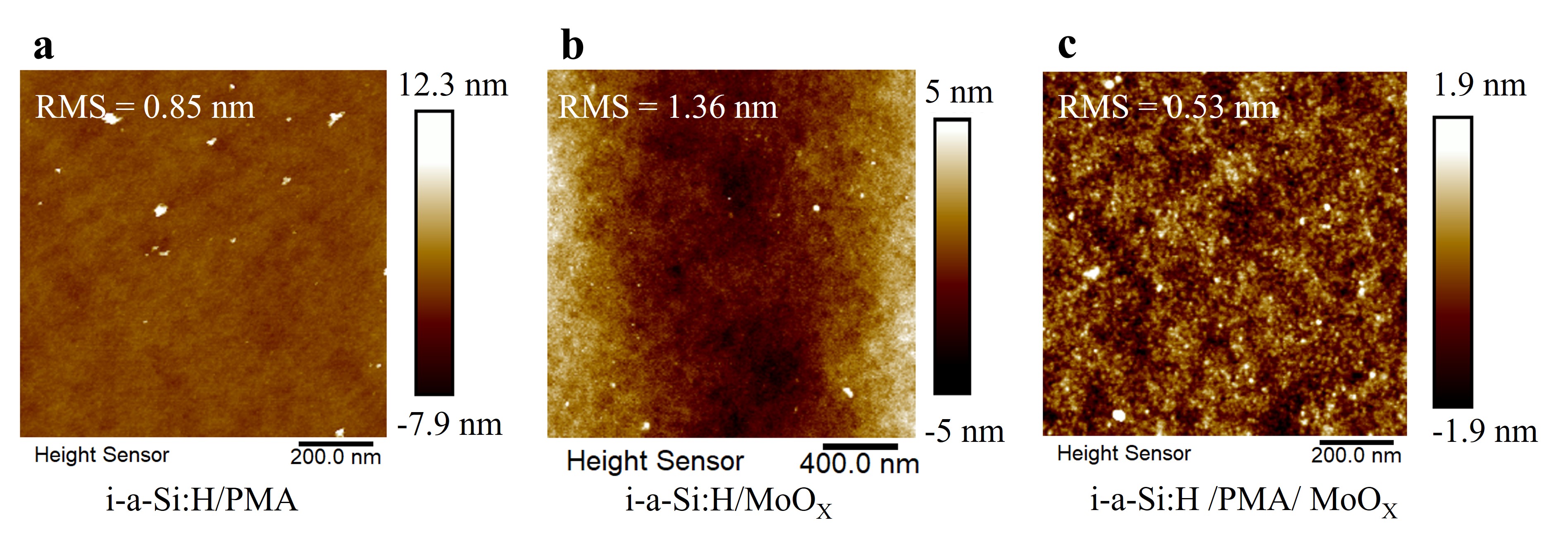


**Fig. S6** Atomic force microscope (AFM) characterization of surface morphology with corresponding root-mean-square (RMS) roughness values for **a** PMA monolayer, **b** MoO_X_ monolayer, and **c** MoO_X_/PMA bilayer.


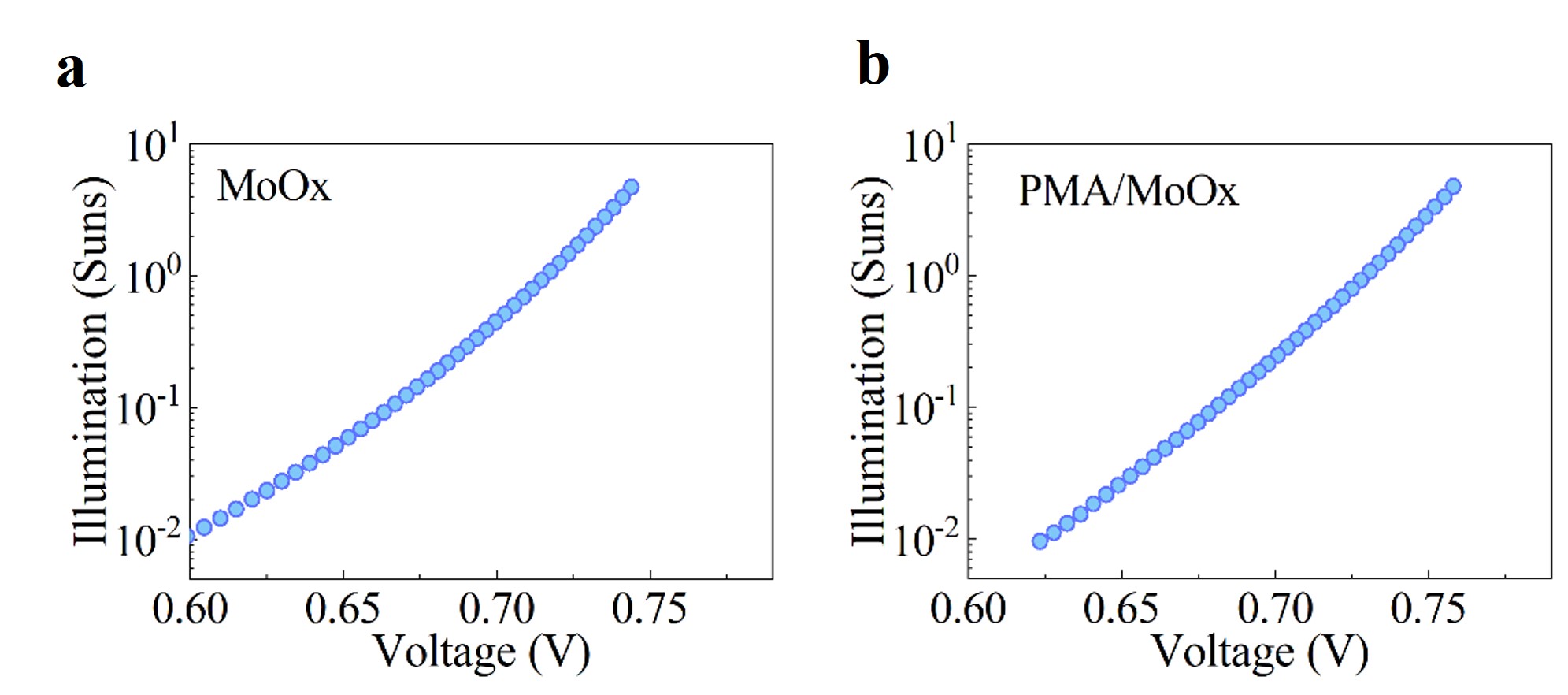


**Fig. S7** Light-intensity-dependent *V*_OC_ of **a** MoO_X_ and **b** PMA/MoO_X_ cells (0.01-1 sun). The superior linearity of the PMA/MoO_X_ device indicates reduced leakage and suppressed interfacial recombination under low-injection conditions.


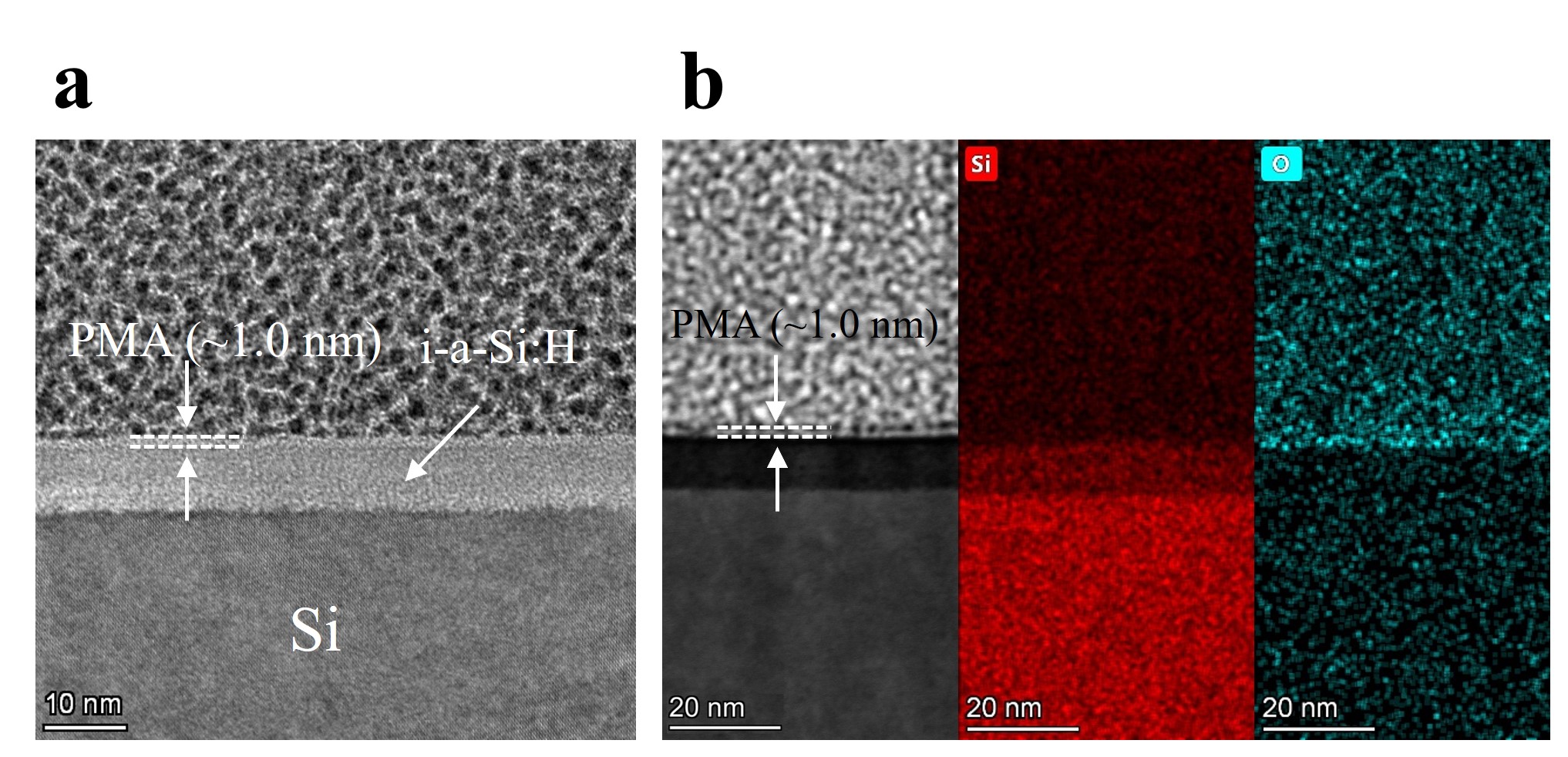


**Fig. S8** **a** Cross-sectional TEM characterization and **b** STEM-EDX mapping of the PMA/i-a‑Si:H/c-Si interface.


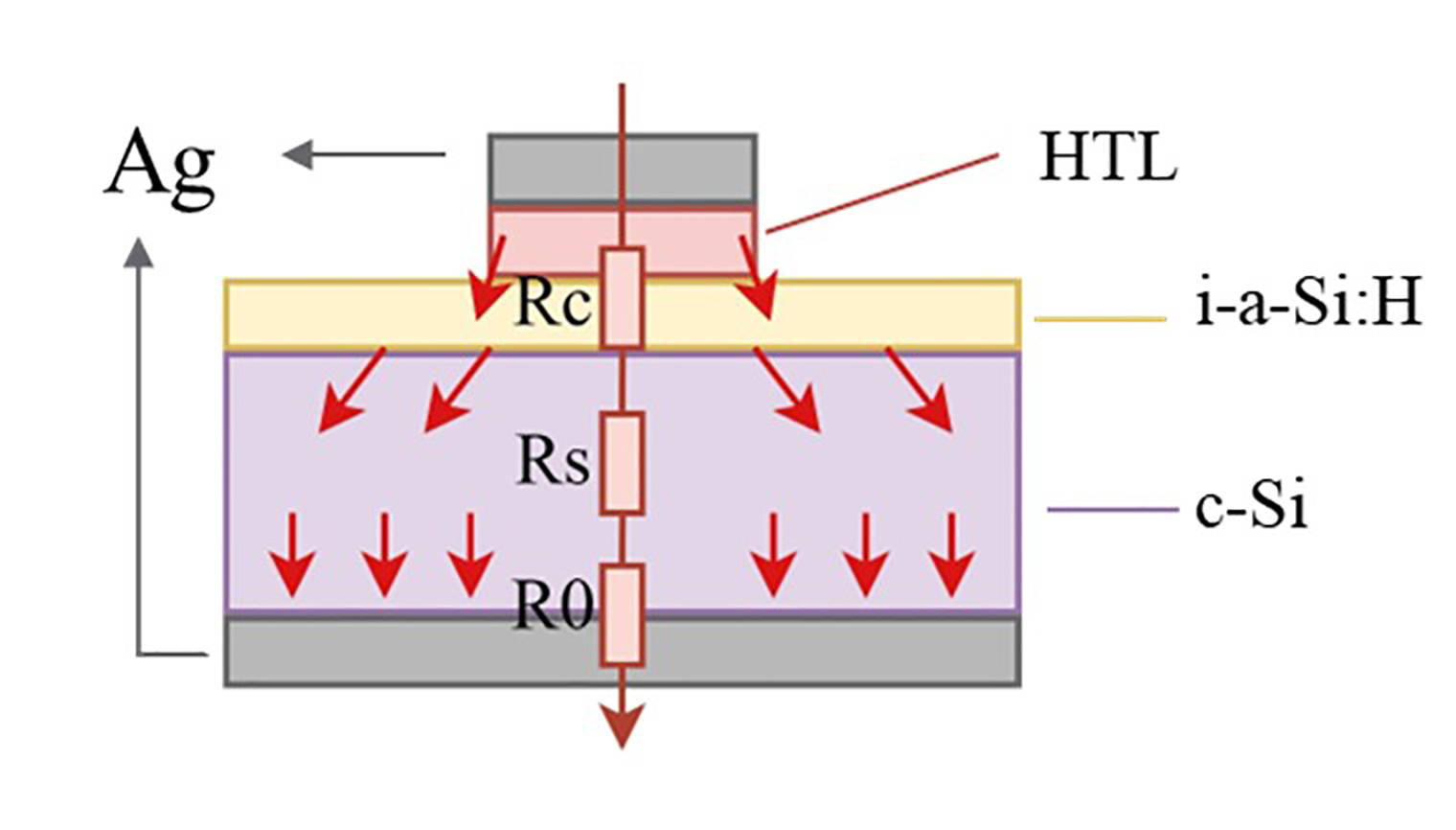


**Fig. S9** 2D cross-sectional current flow lines and equivalent circuit diagrams of the Ag/HTL/i-a-Si:H/c-Si/Ag device, illustrating carrier transport and the origin of series resistance R_S_.


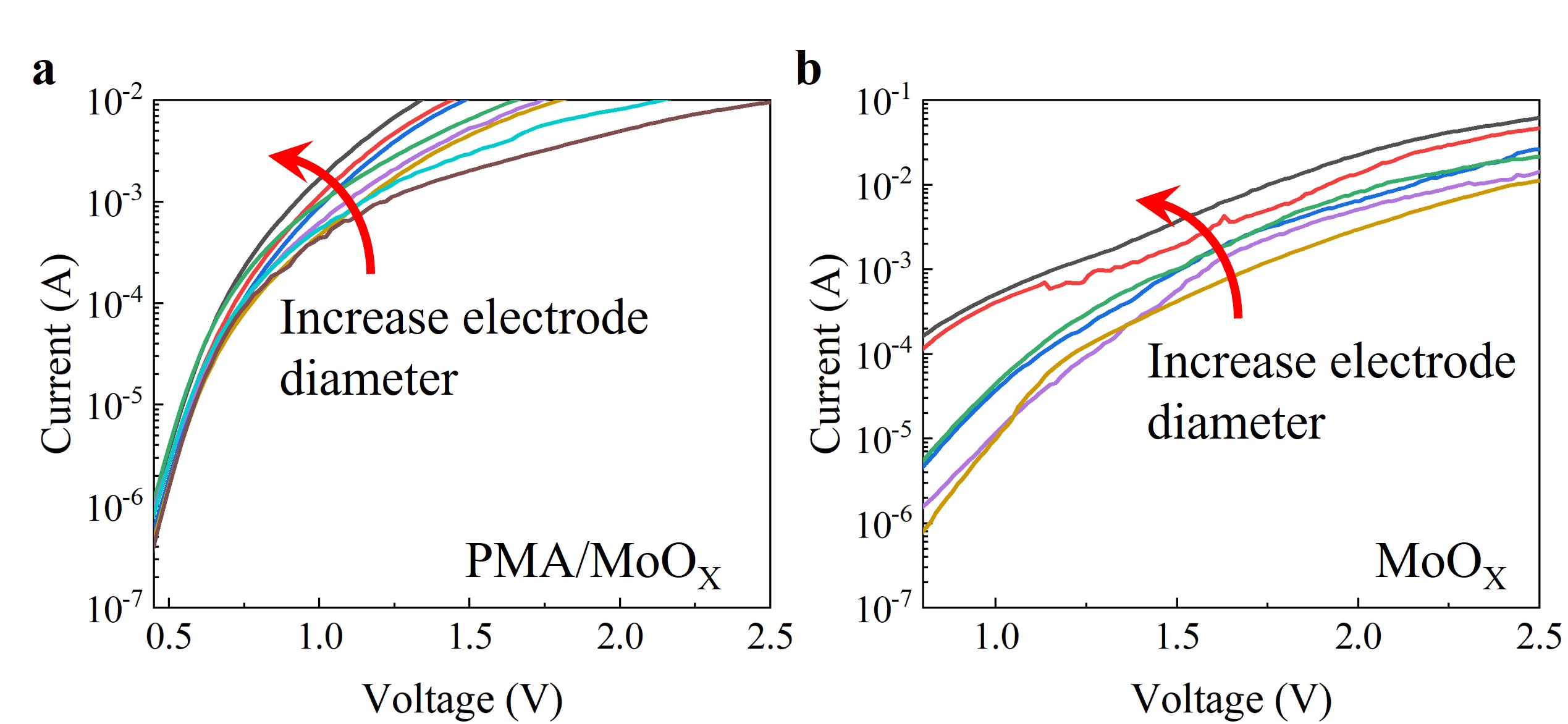


**Fig. S10** Dark I–V characteristics of HTL/i-a-Si:H structures with various electrode diameters for **a** PMA/MoO_X_ and **b** pristine MoO_X_ HTL.


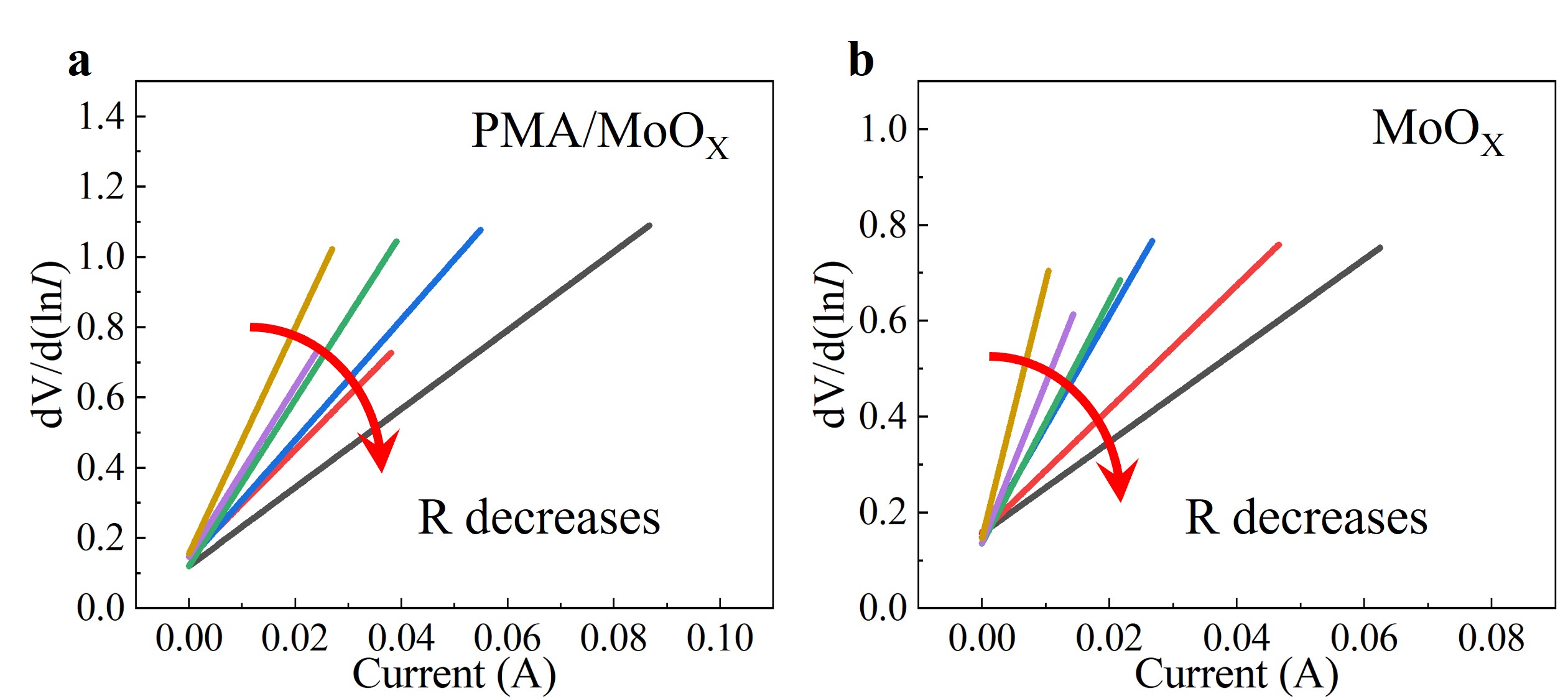


**Fig. S11** Plots of dV/d(lnI) versus I extracted from dark-state IV characteristic curves.

It can be seen from Fig. S9 that the total resistance (*R_T_*) consists of the contact resistance (*R_C_*), spreading resistance (*R_S_*), and residual resistance (*R_0_*), which can be expressed as:

$R_{T}\text{ }=\text{ }R_{C}\text{ }+\text{ }R_{S}\text{ }+\text{ }R_{0}$ (S1)

Among them, *R_0_* is a constant value independent of the diameter of the top electrode, while the spreading resistance *R_S_* has been proven to be negligible. The contact resistance *R_C_* is related to the diameter of the top electrode and is defined as [S1]:

 (S2)

Where d is the diameter of the circular electrode, and *ρ*_c_ is the specific contact resistance. Here, *R_C_* includes not only the contact resistance at the heterojunction interface but also the bulk resistance of the metal electrode and the bulk resistance of the inserted passivation layer i-a-Si:H.

For a typical Schottky contact, we use an extended CSM method to extract the *ρ*_c_. [S2] The value of the *R*_T_ is obtained via the Cheung method and then substituted into the traditional CSM to extract *ρ*_c_. [S3] The equation for obtaining *R*_T_ is:

$\frac{dV}{\text{d}(LnI)}\text{ }=\text{ }\frac{R_{T}}{I}+\frac{\text{nKT}}{\text{q}}\text{ }$ (S3)

Where *V* is the voltage applied to the test sample, *I* is the current flowing through the test sample, *n* is the ideality factor (a constant value), *q* is the unit charge, *k* is the Boltzmann constant, and *T* is the absolute temperature. The electrode diameters used in the experiment are 2.5, 2, 1.6, 1.25, 1, and 0.8 mm, respectively. Here, the device and Ag film were grown continuously via thermal evaporation without exposure to air. The I-V curves of the device tests are shown in Fig. S10a, b. The total resistance *R_T_* can be calculated using the slope of d*V*/d(ln*I*) versus I according to Equation (S3), and the results are shown in Fig. S11a, b.

Subsequently, the calculated *R_T_* values were substituted into the traditional CSM. The detailed steps are as follows: The components of *R_T_* can be known from Equation (S1). Since the spreading resistance *R_S_* has been proven to be negligible, Equation (S1) can be simplified as follows:

$\text{R}\approx R_{T}\text{= }R_{C}+\text{ }R_{0}$ (S4)

Since *R_0_* is mainly a constant (independent of the front electrode area) and, according to Equation (S2), *R_C_* is proportional to 1/S, the resistance *R* has a linear relationship with 1/S, and the slope of this linear relationship is *ρ*_c_. Therefore, the linear curves of *R* versus 1/S can be fitted based on Fig. S11a, b.


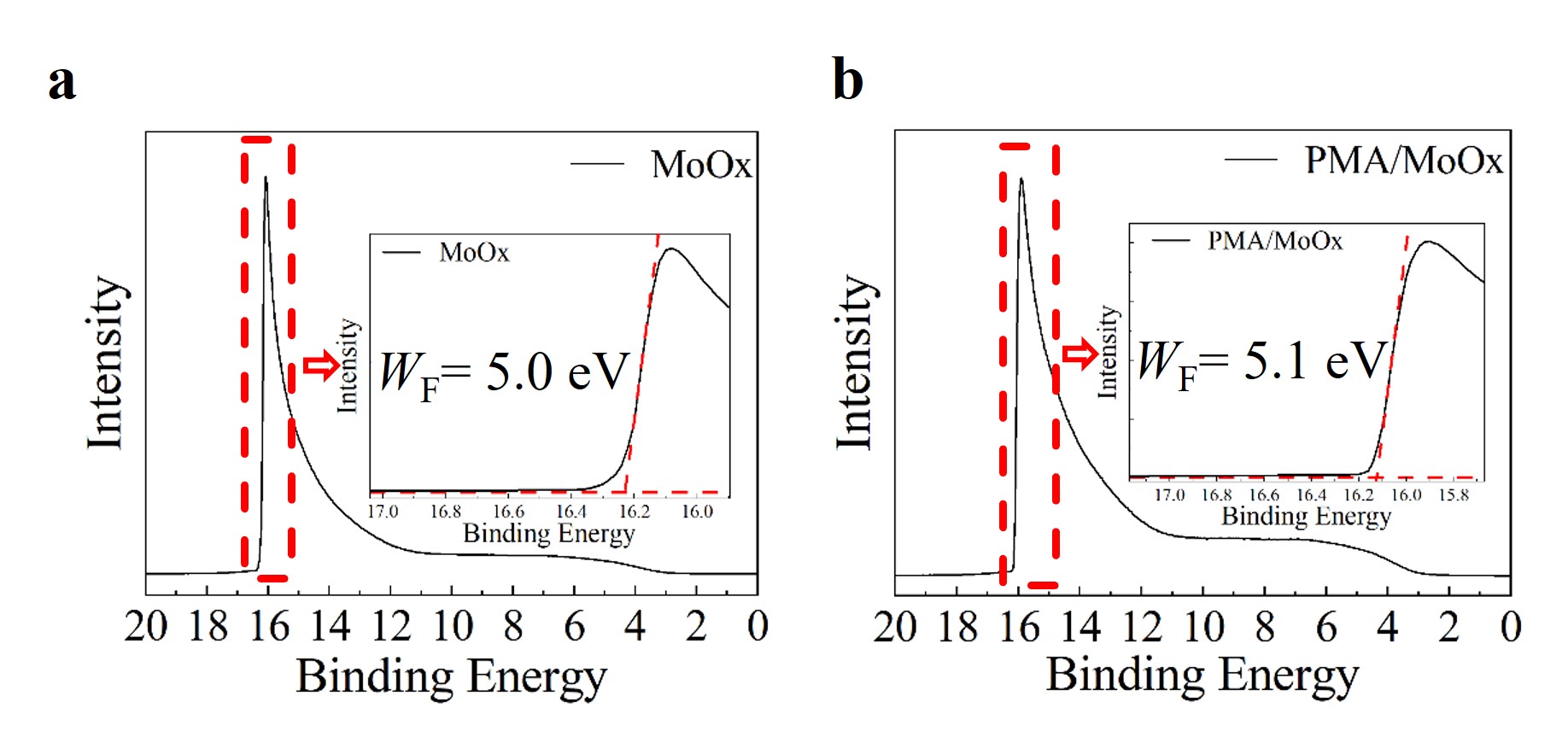


**Fig. S12** UPS spectra of **a** single-layer MoO_X_ and **b** PMA/MoO_X_ films, showing work function (*W*_F_) increases from 5.0 eV to 5.1 eV with PMA.


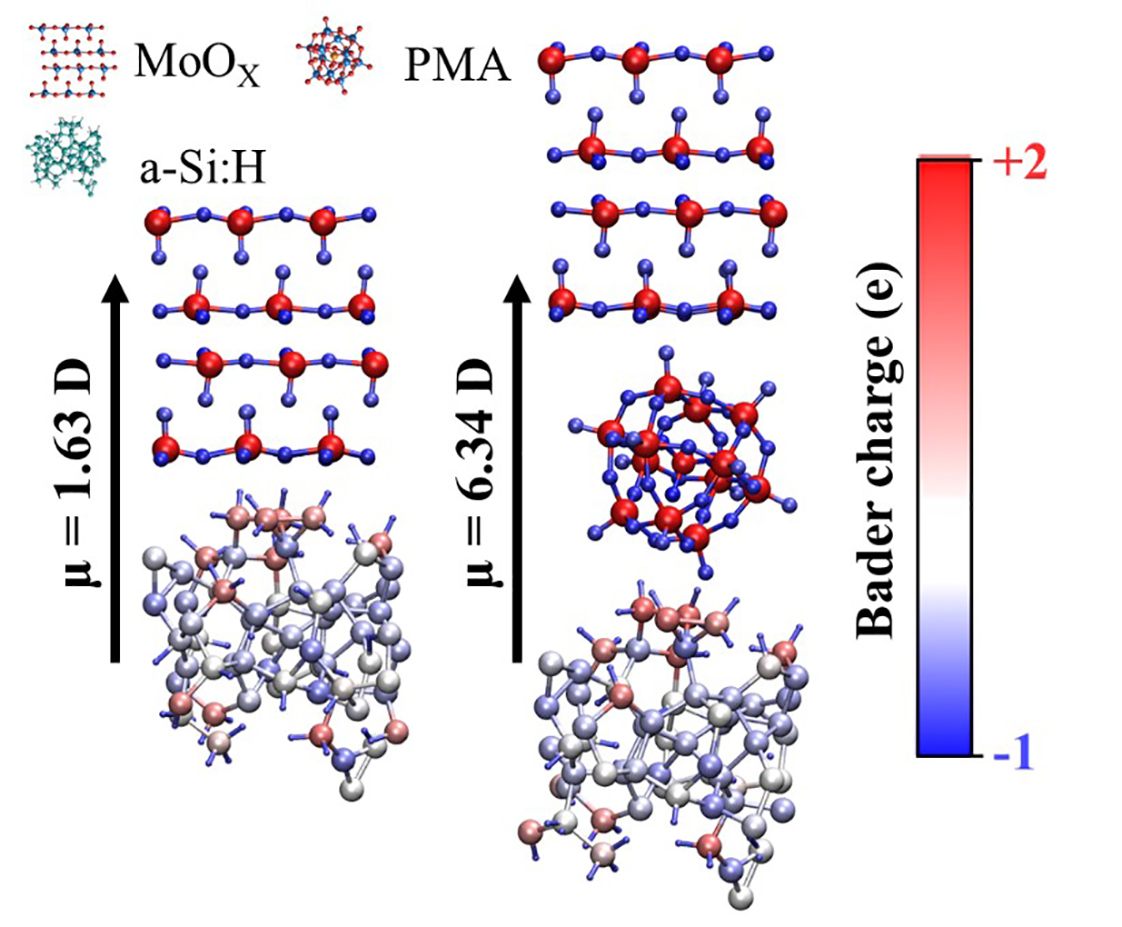


**Fig. S13** DFT‑calculated Bader charge distributions at the MoO_X_/i-a-Si:H interface without (left side) and with (right side) PMA interlayer. The dipole moments (*μ*) obtained from the calculations are marked with each model.


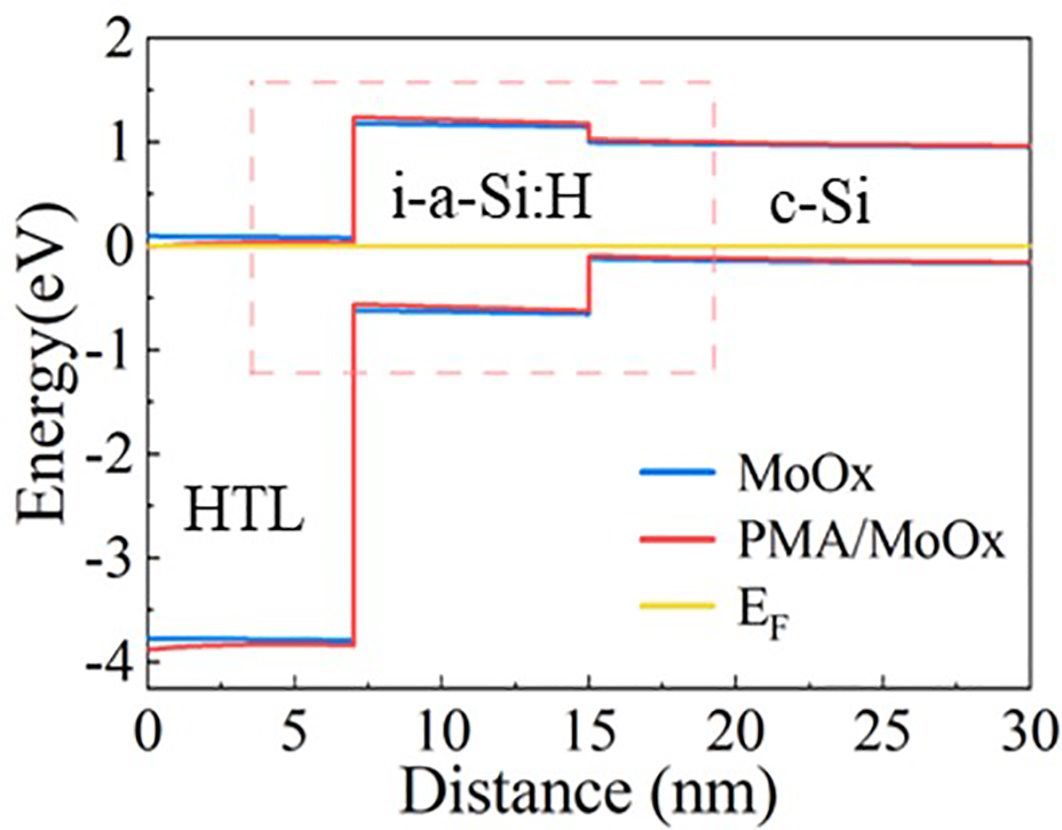


**Fig. S14** Simulated band alignment of the n-Si/i-a-Si:H/HTL stack, comparing the band offset and barrier variation at the MoO_X_ and PMA/ MoO_X_ interfaces.


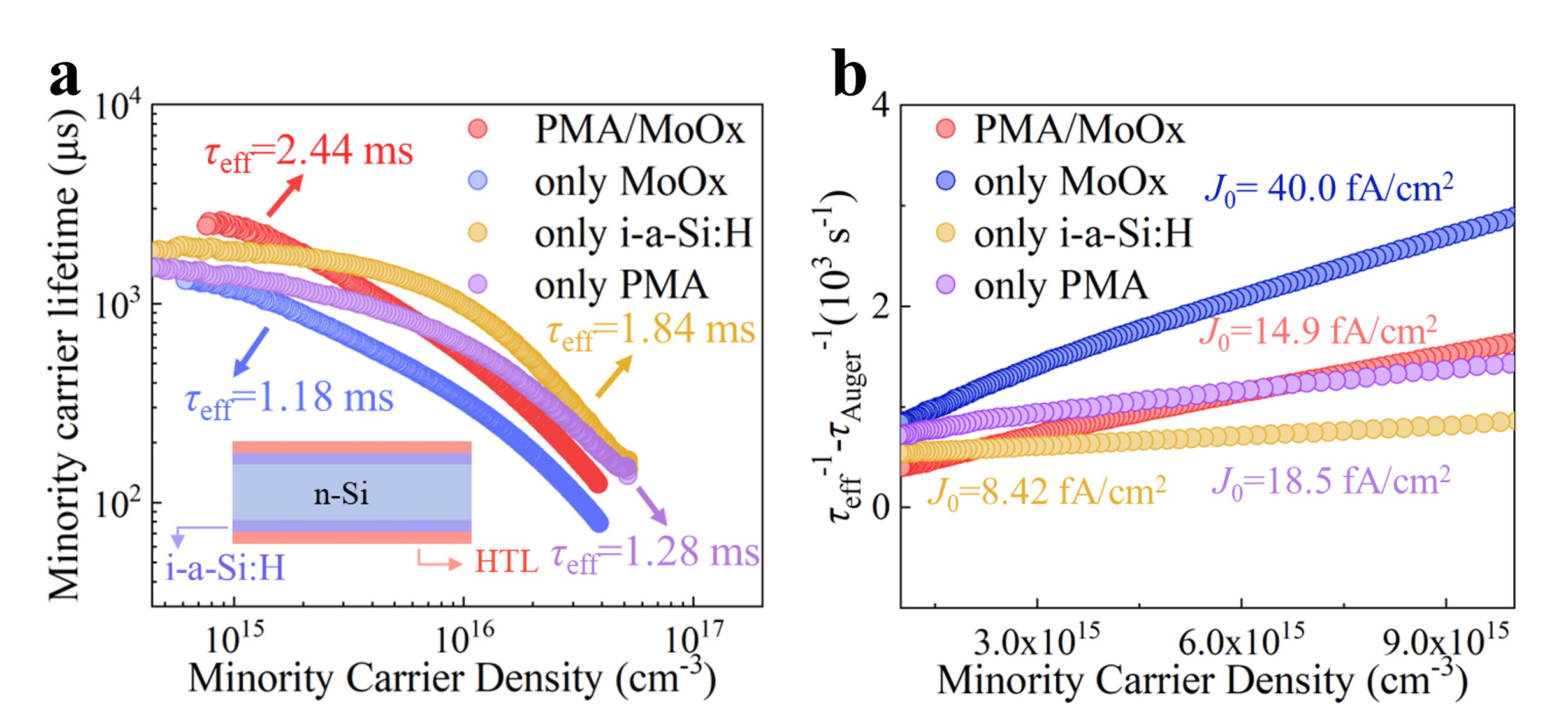


**Fig. S15** Carrier lifetime and interface recombination analysis of different passivation schemes. **a** Effective minority carrier lifetime (*τ_eff_*) as a function of minority carrier density for four structures, including pristine i‑a‑Si:H (yellow), PMA only (purple), MoO_X_ only (blue), and the PMA/MoO_X_ bilayer (red). The highest *τ_eff_* (2.44 ms) is achieved with the PMA/MoO_X_ stack. All *τ*_eff_ values are measured at a carrier density of 1 × 10^15^ cm^-3^. The inset illustrates the corresponding device stack, *n*‑type c-Si/i‑a‑Si:H/hole‑transport layer (HTL). **b** Dependence of (1/*τ_eff_* – 1/*τ_Auger_*) on the minority carrier density, from which the saturation current density (*J_0_*) is extracted. The pristine i‑a‑Si:H layer yields the lowest *J*_0_ overall; among the structures with a hole‑transport layer, the PMA/MoO_X_ bilayer achieves the lowest *J*_0_ (14.9 fA/cm^2^), demonstrating its superior ability to suppress interface recombination while enabling effective hole extraction.

The contact recombination current density (*J*_0_) was extracted from the slope of the linear region in the plot of (1/*τ*_eff_ – 1/*τ*_Auger_) versus excess carrier density (Δ*n*), according to the relation:

(1/*τ*_eff_ – 1/*τ*_Auger_ – 1/*τ*_SRH_) = (2*J*_0_ / (*q* *W* *n_i_*^2^)) Δ*n*  (S5)

where *τ*_SRH_ is the Shockley-Read-Hall recombination lifetime, *q* is the elementary charge, *n_i_* is the intrinsic carrier concentration, and *W* is the wafer thickness. The contribution of *τ*_SRH_ was considered negligible in the high-injection regime used for the extraction, and a standard parameterization of Auger recombination was adopted for *τ*_Auger_.


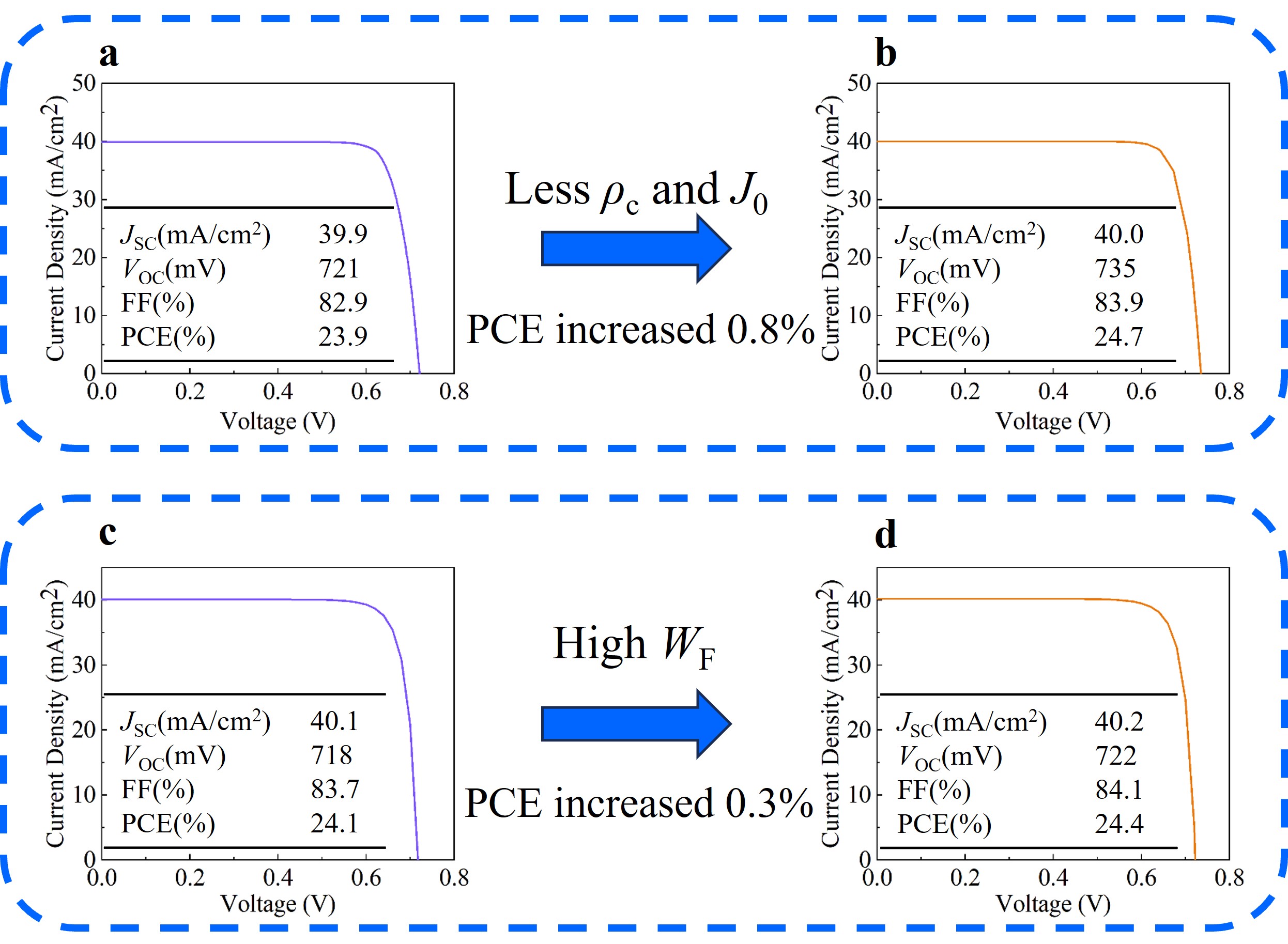


**Fig. S16** Simulation analysis of the device performance enhancement mechanism. **a, b** J-V characteristic curves simulated by Quokka3, showing the effect of *ρ*_c_ and *J*_0_ on device performance. **c, d** J-V characteristic curves simulated by Silvaco, illustrating the impact of increasing the WF of the hole transport layer on device performance. The simulation results indicate that reducing contact resistance and interface recombination, along with optimizing the energy band structure, contributes to the improvement of the power conversion efficiency.

**Table S1** Comparison of Performance Parameters between PMA/MoO_X_ (with Different Concentrations) and MoO_X_ HTL

| Rear strcuture | *J*_SC_ (mA/cm^2^) | *V*_OC_ (mV) | FF (%) | PCE (%) |
| --- | --- | --- | --- | --- |
| 7 nm MoO_X_ | 40.0 | 713 | 83.7 | 23.8 |
| 0.5 mg/ml PMA+7 nm | 39.7 | 716.4 | 79.8 | 22.7 |
| 1 mg/ml PMA+7 nm | 39.9 | 730.99 | 81.09 | 22.86 |
| 2 mg/ml PMA+7 nm | 40.2 | 729.47 | 84.93 | 24.90 |
| 5 mg/ml PMA+7 nm | 39.7 | 727.58 | 80.61 | 23.29 |
| 10mg/ml PMA+2 nm | 39.9 | 698.47 | 70.96 | 19.75 |
| 10mg/ml PMA+7 nm | 37.9 | 727.85 | 73.29 | 20.23 |

**Table S2** Parameters Used for SCAPS Simulation

| Parameters | *n*-Si [4] | MoO_X_ [5] |
| --- | --- | --- |
| Thickness (μm) | 130 | 0.007 |
| Relative dielectric constant | 11.9 | 10 |
| Electron affinity (eV) | 4.0 | 5.13 |
| Band gap (eV) | 1.12 | 3.60 |
| Effective conduction band density (cm^-3^) | 2.8×10^19^ | 1.0×10^19^ |
| Effective valence band density (cm^-3^) | 1.04×10^19^ | 2.84×10^19^ |

**Table S3** Band gap (*E*_g_), electronic affinity (*E*_A_), density of states in conduction band (*N*_C_) and valence band (*N*_V_), donor density (*N*_D_), acceptor density (*N*_A_), dielectric constant (*ε*), as well as mobilities of electron (*μ*_n_) and hole (*μ*_p_) for c-Si, *n*-a-Si:H, *p*-a-Si:H, i-a-Si:H and MoO_X_ used in device simulations

|  | Si | (*n*)a-Si:H | (*p*)a-Si:H | (i)a-Si:H | MoO_X_ |
| --- | --- | --- | --- | --- | --- |
| *E*_g_ (eV) | 1.12 | 1.7 | 1.7 | 1.7 | 3.63^a^ |
| Thickness (nm) | 1.3×10^5^ | 15 | 20 | 5 | 7^a^ |
| *E*_A_ (eV) | 4.10 | 3.8 | 3.8 | 3.8 | 5.11^a^ |
| *ε* | 11.9 | 11.9 | 11.9 | 11.9 | 5.9 [S6] |
| *N*_C_ **(**cm^−3^**)** | 2.8×10^19^ | 2.0×10^20^ | 2.0×10^20^ | 2.0×10^20^ | 2.85×10^19^ [S6] |
| *N*_V_ **(**cm^−3^**)** | 1.0×10^19^ | 2.0×10^19^ | 2.0×10^20^ | 2.0×10^20^ | 2.68×10^19^ [S6] |
| *N*_D_ **(**cm^−3^**)** | 3.0×10^15^ | 1.0×10^19^ | \ | \ | \ |
| *N*_A_ **(**cm^−3^**)** | \ | \ | 1.0×10^19^ | \ | 1×10^19^ [S6] |
| *μ*_n_ **(**cm^2^V^−1^s^−1^**)** | 1350 | 25 | 25 | 20 | 1107 [S6] |
| *μ*_p_ **(**cm^2^V^−1^s^−1^**)** | 450 | 5 | 5 | 4 | 424.6 [S6] |

a: this work

**Table S4** Parameters of defect states on MoO_X_/i-a-Si:H interface [S7]

| Parameter | MoO_X_ |
| --- | --- |
| Defect type | Neutral |
| Capture cross section electrons (cm^2^) | 10^−19^ |
| Capture cross-section holes (cm^2^) | 10^−19^ |
| Energy with respect to Reference (eV) | 0.06 |
| Total defect density (cm^-2^) | 10^10^ |

**Supplementary References**

1. R. H. Cox, H. Strack. Ohmic contacts for gaas devices. Solid-State Electron. **10**(12), 1213-1218 (1967). <https://doi.org/10.1016/0038-1101(67)90063-9>
2. W. Wang, H. Lin, Z. H. Yang, Z. L. Wang, J. J. Wang et al., An expanded cox and strack method for precise extraction of specific contact resistance of transition metal oxide/n-silicon heterojunction. IEEE J. Photovoltaics. **9**(4), 1113-1120 (2019). <https://doi.org/10.1109/Jphotov.2019.2917386>
3. T. Morimoto, M. Chiba. Comment on ‘‘galvanomagnetic luminescence of indium antimonide’’[appl. Phys. Lett. 4 7, 1330 (1985)]. Appl. Phys. Lett. **49**(9), 537-537 (1986). <https://doi.org/10.1063/1.97104>
4. B. K. Mondal, S. K. Mostaque, M. A. Rashid, A. Kuddus, H. Shirai et al., Effect of CdS and In_3_Se_4_ bsf layers on the photovoltaic performance of PEDOT:PSS /n-si solar cells: Simulation based on experimental data. Superlattices Microstruct. **152**(106853 (2021). <https://doi.org/10.1016/j.spmi.2021.106853>
5. L. Li, G. L. Du, Y. Y. Lin, X. Zhou, Z. Y. Gu et al., NiOx/MoOx bilayer as an efficient hole-selective contact in crystalline silicon solar cells. Cell Rep. Phys. Sci. **2**(12), 100684 (2021). <https://doi.org/10.1016/j.xcrp.2021.100684>
6. M. Q. Khokhar, K. Mallem, X. Fan, Y. Kim, S. Q. Hussain et al., Experimental and numerical simulation of molybdenum oxide films with wide bandgap and high work function for carrier-selective contact solar cells. Ecs Journal of Solid State Science and Technology. **11**(8), 085001 (2022). [https://doi.org/](https://doi.org/10.1016/j.xcrp.2021.100684)10.1149/2162-8777/ac8372
7. A. Ait Abdelkadir, E. Oublal, M. Sahal, B. M. Soucase, A. Kotri et al., Numerical simulation and optimization of n-Al-ZnO/n-CdS/p-CIGS/p-si/p-MoOx/Mo tandem solar cell. Silicon. **15**(5), 2125-2135 (2023). https://doi.org/10.1007/s12633-022-02144-1
